# A 5′, 8-cyclo-2′-deoxypurine lesion induces trinucleotide repeat deletion via a unique lesion bypass by DNA polymerase β

**DOI:** 10.1093/nar/gku1239

**Published:** 2014-11-26

**Authors:** Meng Xu, Yanhao Lai, Zhongliang Jiang, Michael A. Terzidis, Annalisa Masi, Chryssostomos Chatgilialoglu, Yuan Liu

**Affiliations:** 1 Department of Chemistry and Biochemistry, Florida International University, 11200 SW, 8th Street, Miami, FL 33199, USA; 2 Biomolecular Sciences Institute, School of Integrated Sciences and Humanities, Florida International University, 11200 SW, 8th Street, Miami, FL 33199, USA; 3 ISOF, Consiglio Nazionale delle Ricerche, Via P. Gobetti 101, 40129 Bologna, Italy; 4 Institute of Nanoscience and Nanotechnology, N.C.S.R. ‘Demokritos’, 15341 Agia, Paraskevi, Athens, Greece

## Abstract

5′,8-cyclo-2′-deoxypurines (cdPus) are common forms of oxidized DNA lesions resulting from endogenous and environmental oxidative stress such as ionizing radiation. The lesions can only be repaired by nucleotide excision repair with a low efficiency. This results in their accumulation in the genome that leads to stalling of the replication DNA polymerases and poor lesion bypass by translesion DNA polymerases. Trinucleotide repeats (TNRs) consist of tandem repeats of Gs and As and therefore are hotspots of cdPus. In this study, we provided the first evidence that both (5′R)- and (5′S)-5′,8-cyclo-2′-deoxyadenosine (cdA) in a CAG repeat tract caused CTG repeat deletion exclusively during DNA lagging strand maturation and base excision repair. We found that a cdA induced the formation of a CAG loop in the template strand, which was skipped over by DNA polymerase β (pol β) lesion bypass synthesis. This subsequently resulted in the formation of a long flap that was efficiently cleaved by flap endonuclease 1, thereby leading to repeat deletion. Our study indicates that accumulation of cdPus in the human genome can lead to TNR instability via a unique lesion bypass by pol β.

## INTRODUCTION

**Figure 1.**
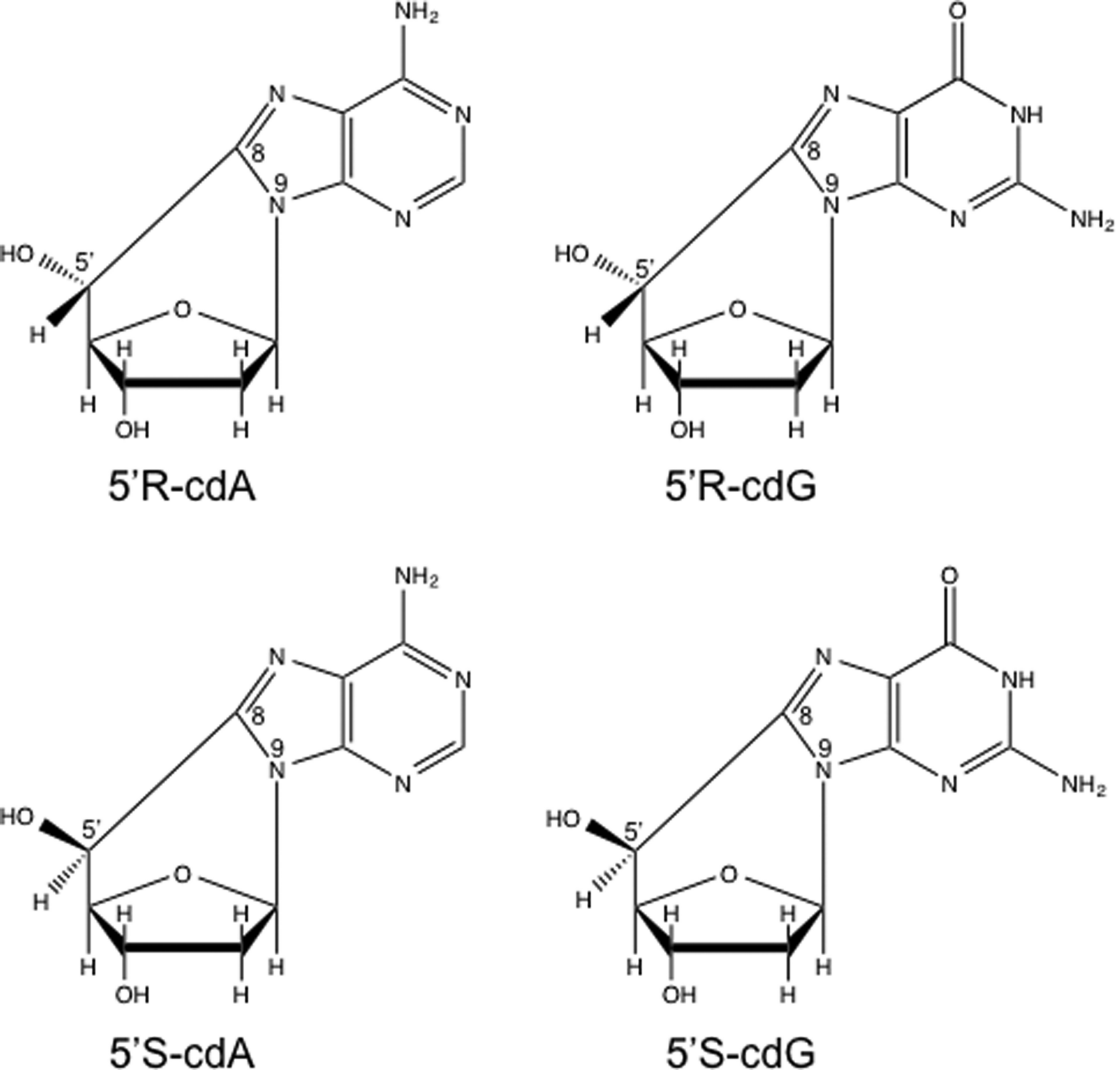
The structure of 5′,8-cyclo-2′-deoxynucleosides.

Reactive oxygen species (ROS) generated from environmental stress such as ionizing radiation and endogenous oxidative stress from energy metabolism can result in more than 30 types of oxidized DNA base lesions in the human genome (1,2). Among them, 5′,8-cyclo-2′-deoxypurines (cdPus), including 5′,8-cyclo-2′-deoxyadenosine (cdA) and 5′,8-cyclo-2′-deoxyguanosine (cdG) (Figure 1), are the ones that can induce DNA structure abnormality (1,3–5). cdPu lesions contain an extra covalent bond formed between the C5 of the 2′-deoxyribose and the C8 of the purine. cdPus occur in two diastereomeric forms with a 5′R or 5′S configuration (Figure 1). The lesions are frequently detected in the genomic DNA of mammals and other organisms (6–8). It is estimated that 180–320 cdPus/cell can be produced in fetal and postnatal rat liver per day (6). Because the extra covalent bond of a cdPu prevents cleavage of the glycosidic bond by DNA glycosylases (9–11), the lesion can only be repaired by nucleotide excision repair (NER) (9,12,13). However, the efficiency of removing a cdPu by NER is 2- to 4-fold less than that of removing other bulky lesions such as a cis-B[α]P-N^2^-dG adduct (12). This leads to the accumulation of a high level of cdPu lesions in genomic DNA, resulting in blockage of DNA synthesis by human pol δ/ϵ (13), replication fork stalling and inefficient lesion bypass DNA synthesis by translesion DNA polymerases (14–16). This can subsequently lead to the accumulation of DNA strand breaks that can ultimately cause cell death. Because the bypass of a cdPu by translesion DNA polymerases, such as pol ζ and pol κ, can also cause A to T and G to T transversions as well as a G to A transition (14), accumulation of cdPus in the genome may result in mutagenesis and cell death, which are associated with aging, cancer and neurodegeneration (7,17–19).

Trinucleotide repeats (TNRs) are highly polymorphic in the human genome and can be expanded or deleted during DNA replication, DNA repair and gene transcription (20–27). TNR expansion is associated with more than 40 neurodegenerative diseases such as Huntington's disease (21,28,29). TNR deletion in the androgen receptor gene is associated with ovarian and prostate cancers (30–32). It has been found that the formation of non-B form DNA secondary structures, such as hairpins, triplexes and tetraplexes, is responsible for TNR expansion and deletion during DNA metabolism and gene transcription (33–38). The secondary structures formed in a newly synthesized or damaged strand of genomic DNA usually lead to TNR expansion via a variety of mechanisms during DNA replication and repair (37–46). In contrast, TNR secondary structures formed in the template strand usually cause TNR deletion via skip over of the structures by DNA polymerases (26,27,38).

TNRs contain multiple adenosines and guanines. This makes them hotspots for the formation of oxidized base lesions, including cdPu lesions. Previous studies have shown that oxidative DNA damage, 8-oxoguanine (8-oxoG) and its repair in a TNR tract are associated with TNR expansion (47–49). An increased level of 8-oxoG in the brain of HD transgenic mouse models and the germ cells of a fragile X syndrome mouse model was correlated with CAG and CGG repeat expansions (47–49). We have shown that an environmental oxidative DNA damaging agent, potassium bromate, can induce both expansion and deletion of CTG repeats in human kidney cells (26). We further demonstrated that TNR instability was initiated by the formation of a hairpin in the damaged or the template strand at different locations in a TNR tract and that this instability is mediated by the coordination between DNA polymerase β (pol β) and flap endonuclease 1 (FEN1) during DNA base excision repair (BER) (26,27,43). Moreover, we have recently discovered that BER of a hairpin loop can also lead to removal of a hairpin (50), thereby preventing and attenuating repeat expansion. This indicates that an oxidized base lesion can modulate TNR instability through its repair on the damaged strand in a damage location-dependent manner. However, it remains unknown if and how an oxidized DNA base lesion such as a cdPu that readily accumulates in the template strand of a TNR tract can affect TNR instability during DNA replication and repair. Although cdPus occur in genomic DNA at a lower frequency than 8-oxoG (6), the inefficient removal of the lesions by NER can lead to a much higher level of accumulation in the genome than that of 8-oxoG, which is efficiently removed by BER. Thus, cdPu lesions may induce TNR instability as efficiently as 8-oxoGs.

Because pol β DNA synthesis plays a crucial role in mediating TNR instability (26,27,51) and it can bypass various types of base damage including an AP site, cisplatin- and oxaliplatin-DNA adducts, a thymine dimer and a BaP-dG adduct during BER and DNA replication (52–62), we asked whether pol β DNA synthesis can result in TNR instability via bypassing a cdPu lesion during both DNA replication and BER. We hypothesized that pol β bypasses a cdPu lesion located in a TNR tract during DNA replication and BER, resulting in the synthesis of extra units of TNRs or skip-over of repeats, which subsequently causes TNR instability. To test this hypothesis, we examined pol β DNA synthesis for bypassing a cdA in the template strand of a (CAG)_10_ repeat tract and determined the effects of the base lesion on CTG repeat instability. We provide the first evidence that a cdA in a CAG repeat tract can be bypassed by pol β skip-over of a loop structure that contains the lesion. This preferentially results in CTG repeat deletion.

## MATERIALS AND METHODS

### Materials

DNA oligonucleotides containing a 5′S-cdA or 5′R-cdA were synthesized and purified by High-performance liquid chromatography (HPLC) according to the procedures described previously (63). All other oligonucleotides were purchased from Integrated DNA Technologies (IDT Inc., Coralville, IA, USA). The radionuclides [γ-^32^P] ATP (6000 mCi/mmol) and Cordycepin 5′-triphosphate 3′-[α-^32^P] (5000 mCi/mmol) were purchased from PerkinElmer Inc. (Boston, MA, USA). Micro Bio-Spin 6 chromatography columns were from Bio-Rad (Hercules, CA, USA). Deoxynucleoside 5′-triphosphates (dNTPs) were from Fermentas (Glen Burnie, MD, USA). S1 Nuclease was from Promega (Madison, WI, USA). All other standard chemical reagents were purchased from Sigma-Aldrich (St. Louis, MO, USA) and Thermo Fisher Scientific (Pittsburgh, PA, USA). Purified pol β, FEN1 and DNA ligase I (LIG I) were generous gifts from Dr Samuel H. Wilson at the National Institute of Environmental Health Sciences (NIEHS)/National Institutes of Health, Research Triangle Park, NC.

### Oligonucleotide substrates

Substrates containing a template strand with a dA, or 5′S-cdA, or 5′R-cdA located at the fifth repeat unit of (CAG)_10_ repeats counted from the 5′-end, were designed to mimic DNA replication or BER intermediates without or with a template cdA lesion. The downstream primers of the substrates with (CTG)_9_, (CTG)_5_ repeats or (CTG)_1_, contained a 5′-phosphate or a tetrahydrofuran (THF) residue with a 5′-phosphate, an abasic site analog. The corresponding upstream primers contained a 3′-(CTG)_1_, (CTG)_5_ or (CTG)_9_ repeats, respectively. The substrates were used to represent replication and BER intermediates with a 1-nt gap opposite a template cdA located at different positions of a (CAG)_10_ repeat tract. Substrates with a 1-nt gap were constructed by annealing the upstream and downstream primers to their template strands at a molar ratio of 1:1:1.5. Substrates representing an open template during DNA lagging strand synthesis were constructed by annealing the upstream primer containing (CTG)_5_ repeats with the template strands with or without cdA at a molar ratio of 1:1.5. Substrates representing an intact double strand DNA with or without a template cdA in a (CAG)_10_ repeat tract were constructed by annealing the template strands with their complimentary strand at a molar ratio of 1:1.5. Substrates were radiolabeled at the 5′-end of the upstream primers or the 5′-end of the template strands, or the 3′-end of the downstream primers for measuring various types of enzymatic activities. The sequences of oligonucleotide substrates are listed in Supplementary Table S1. DNA size markers that correspond to the repaired or ligated products from BER or DNA lagging strand maturation, FEN1 cleavage products and S1 Nuclease cleavage products were synthesized and purified by urea-denaturing polyacrylamide gel electrophoresis (PAGE).

### *In vitro* BER and Okazaki fragment maturation assays

*In vitro* BER of an intermediate with a 1-nt gap at different locations opposite a template cdA lesion in the context of (CAG)_10_ repeats was reconstituted with purified pol β, FEN1, LIG I and substrates containing a template dA or cdA and a 5′-phosphorylated THF residue in the downstream primers. Okazaki fragment maturation was reconstituted with purified pol β, FEN1, LIG I and substrates (25 nM) containing a template dA or cdA and a 5′-phosphorylated downstream primer or without a downstream primer. Substrates were ^32^P-labeled at the 5′-end of the upstream primers. Ten microliters of reaction were reconstituted with the indicated concentrations of substrates and the enzymes in buffer that contained 50 mM Tris pH 7.5, 5 mM MgCl_2_, 2 mM adenosine triphosphate (ATP) and 50 μM dNTPs. Reaction mixtures were assembled on ice and incubated at 37°C for 30 min. Reactions were then terminated by transfer to 95°C for 10 min in buffer containing 95% formamide and 10 mM ethylenediaminetetraacetic acid. Substrates and products were separated by 15% urea-denaturing PAGE and detected by PhosphorImagery. Experiments were repeated at least three times.

### Enzymatic activity assays

Pol β DNA synthesis was measured using substrates that mimic DNA replication and BER intermediates with a template strand that contained (CAG)_10_ repeats with or without a cdA. DNA synthesis was measured at 37°C in a 10 μl reaction mixture that contained buffer with 50 mM Tris pH 7.5, 5 mM MgCl_2_ and 50 μM dNTPs. FEN1 flap cleavage activity was examined in the same buffer with 5 mM MgCl_2_ and 50 μM dNTPs in the absence or presence of increasing concentrations of pol β at 37°C for 30 min. Substrates and products were separated by 15% urea-denaturing PAGE and detected by PhosphorImagery. Experiments were repeated at least three times.

### Probing of secondary structures by S1 nuclease digestion

Formation of CAG repeat hairpin or loop structures in the template strand was probed by incubating 0.15 U or 0.05 U S1 Nuclease with 25 nM substrates that contained (CAG)_10_ repeats with or without a cdA embedded in the fifth CAG in the template strands in the presence or absence of pol β. Substrates were pre-incubated with 5 nM pol β at 37°C for 30 min to generate a pol β lesion bypass synthesis intermediate. The 10 μl reaction mixture was assembled in reaction buffer containing 50 mM sodium acetate (pH 4.5), 280 mM NaCl and 4.5 mM ZnSO_4_. The reaction was incubated at 37°C for 3, 5 and 10 min, and subsequently subject to protease K digestion at 55°C for 30 min. Substrates and products were separated by 15% urea-denaturing PAGE and detected by PhosphorImagery. All experiments were repeated at least three times.

## RESULTS

### A cdA in the template strand of a (CAG)_10_ repeat tract can be bypassed by pol β during BER and Okazaki fragment maturation

**Figure 2.**
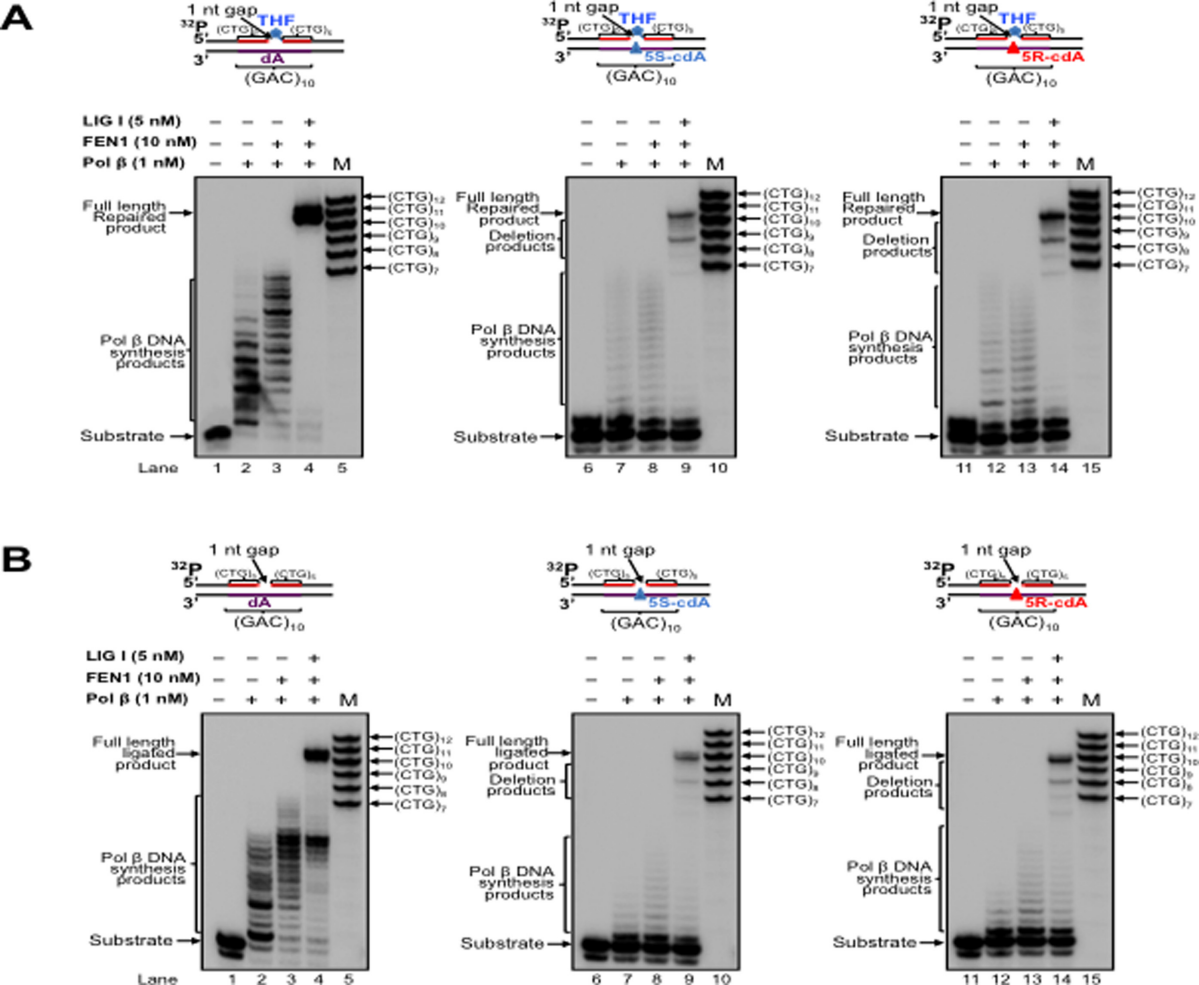
CTG repeat deletion through pol β bypass of a cdA in a (CAG)_10_ tract opposite a 1-nt gap during BER and DNA replication. (**A**) Pol β bypass of a cdA in a (CAG)_10_ repeat tract during BER was examined with substrates having a 1-nt gap opposite a template dA, or 5′S-cdA or 5′R-cdA located at the fifth CAG of the (CAG)_10_ repeat-containing template strand and the downstream primer containing a 5′-phosphorylated THF residue. Lanes 1, 6 and 11 correspond to substrates only. Lanes 2–3, 7–8 and 12–13 correspond to reaction mixtures with 1-nM pol β in the absence or presence of 10-nM FEN1. Lanes 4, 9 and 14 correspond to reaction mixtures with 1-nM pol β, 10-nM FEN1 and 5-nM LIG I. Lanes 5, 10 and 15 correspond to a series of synthesized size markers (M) for illustrating the size of repaired products. (**B**) Pol β bypass of a cdA in the context of CAG repeats during Okazaki fragment maturation was examined with substrates containing a 1-nt gap opposite a template dA, or 5′S-cdA, or 5′R-cdA located in the fifth CAG of the (CAG)_10_ repeat-containing template and the downstream primer with a 5′-phosphate. Lanes 1, 6 and 11 correspond to substrates only. Lanes 2–3, 7–8 and 12–13 correspond to reaction mixtures with 1-nM pol β in the absence or presence of 10-nM FEN1. Lanes 4, 9 and 14 correspond to reaction mixtures with 1-nM pol β, 10-nM FEN1 and 5-nM LIG I. Lanes 5, 10 and 15 correspond to a series of synthesized size markers (M). Substrates were radiolabeled at the 5′-end of the upstream primer.

**Figure 3.**
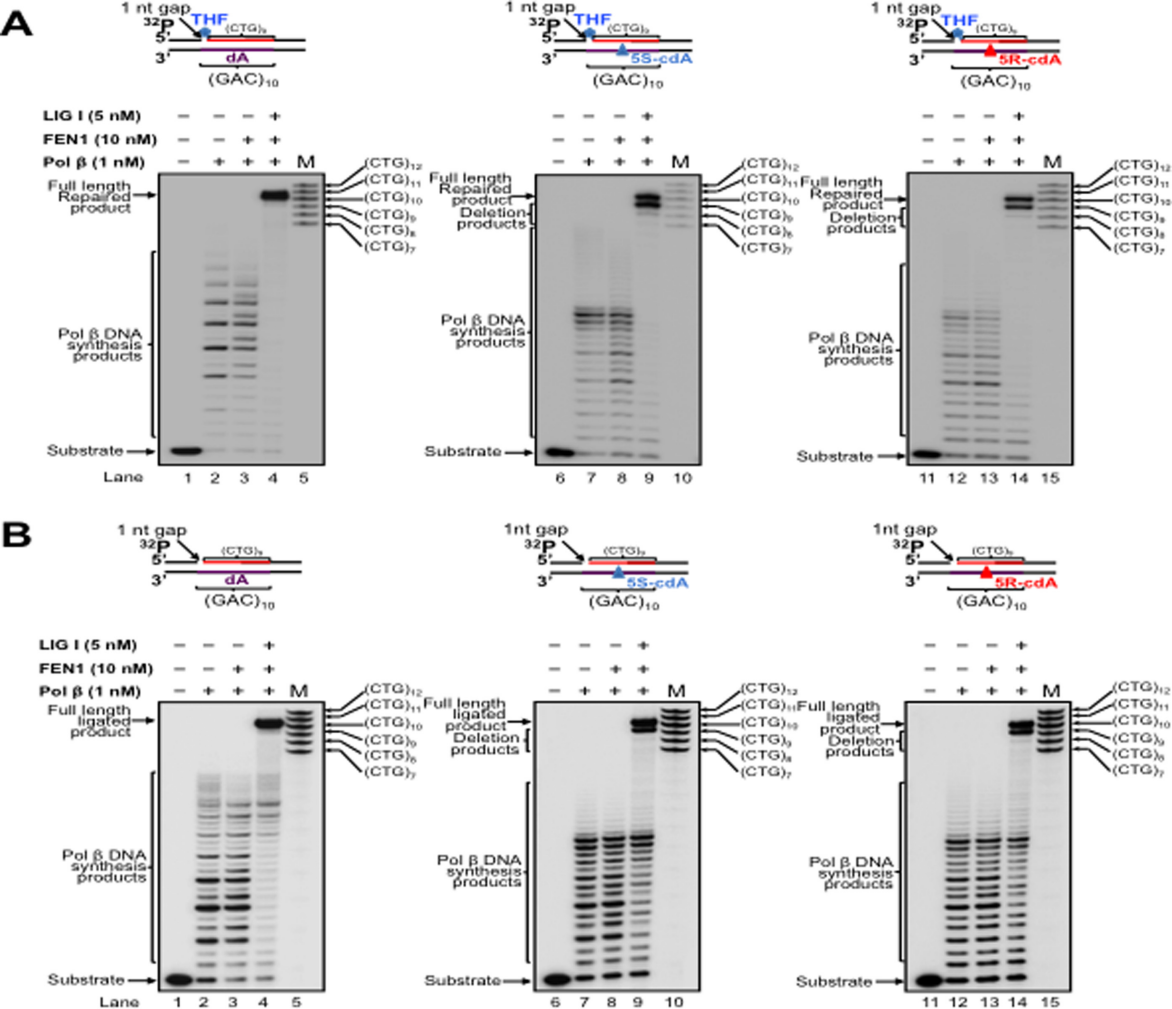
CTG repeat deletion via pol β bypass of a cdA in a (CAG)_10_ tract located downstream of a 1-nt gap during BER and DNA replication. (**A**) Pol β bypass of a template cdA located downstream of a 1-nt gap during BER was examined with the substrates containing a 1-nt gap upstream of a dA, or 5′S-cdA, or 5′R-cdA and the downstream primer containing a 5′-phosphorylated THF residue. (**B**) Pol β bypass of a cdA in CAG repeats located downstream of a 1-nt gap during Okazaki fragment maturation was examined with the substrates containing a 1-nt gap upstream of a template dA, or 5S-cdA, or 5R-cdA at the fifth CAG of (CAG)_10_ repeat template and the 5′-phosphorylated downstream primer. In both panels, lanes 1, 6 and 11 correspond to substrates only. Lanes 2–3, 7–8 and 12–13 correspond to reaction mixtures with 1-nM pol β in the absence or presence of 10-nM FEN1. Lanes 4, 9 and 14 correspond to reaction mixtures with 1-nM pol β, 10-nM FEN1 and 5-nM LIG I. Lanes 5, 10 and 15 correspond to a series of synthesized size markers (M).

**Figure 4.**
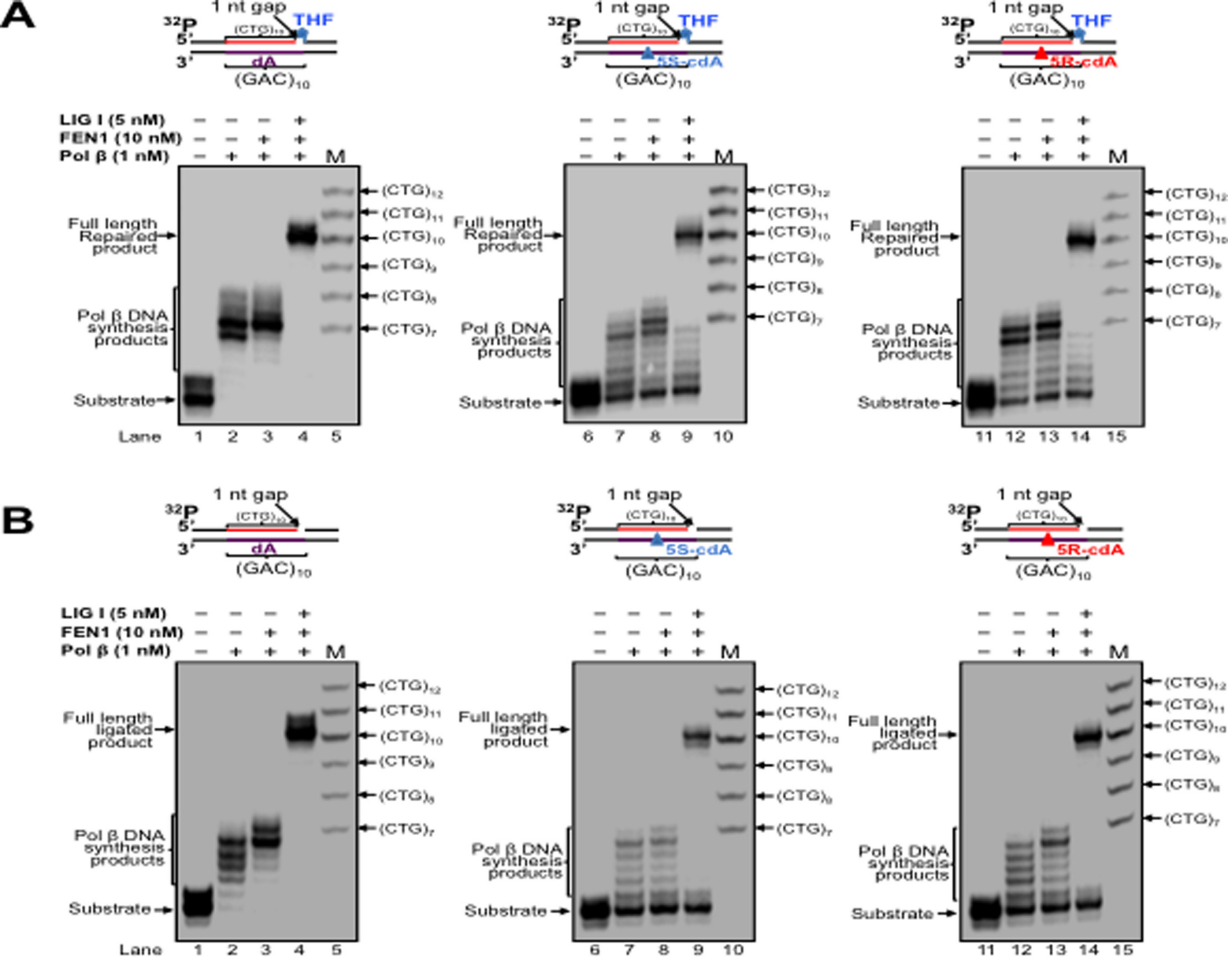
Pol β DNA synthesis with a template cdA located upstream of a 1-nt gap during BER and DNA replication. (**A**) Pol β DNA synthesis with a template cdA located upstream of a 1nt-gap during BER was examined with the substrates containing a template dA, or 5′S-cdA, or 5′R-cdA located upstream of a 1-nt gap and a downstream primer with a 5′-phosphorylated THF residue. (**B**) Pol β DNA synthesis with a template cdA located downstream of a 1-nt gap during Okazaki fragment maturation was examined with the substrates with a 1-nt gap located downstream of a template dA, or 5′S-cdA, or 5′R-cdA and a 5′-phosphorylated downstream primer. In both panels, lanes 1, 6 and 11 correspond to substrates only. Lanes 2–3, 7–8 and 12–13 correspond to reaction mixtures with 1-nM pol β in the presence or absence of 10-nM FEN1. Lanes 4, 9 and 14 correspond to reaction mixtures with 1-nM pol β, 10-nM FEN1 and 5-nM LIG I. Lanes 5, 10 and 15 correspond to a series of synthesized size markers (M).

**Figure 5.**
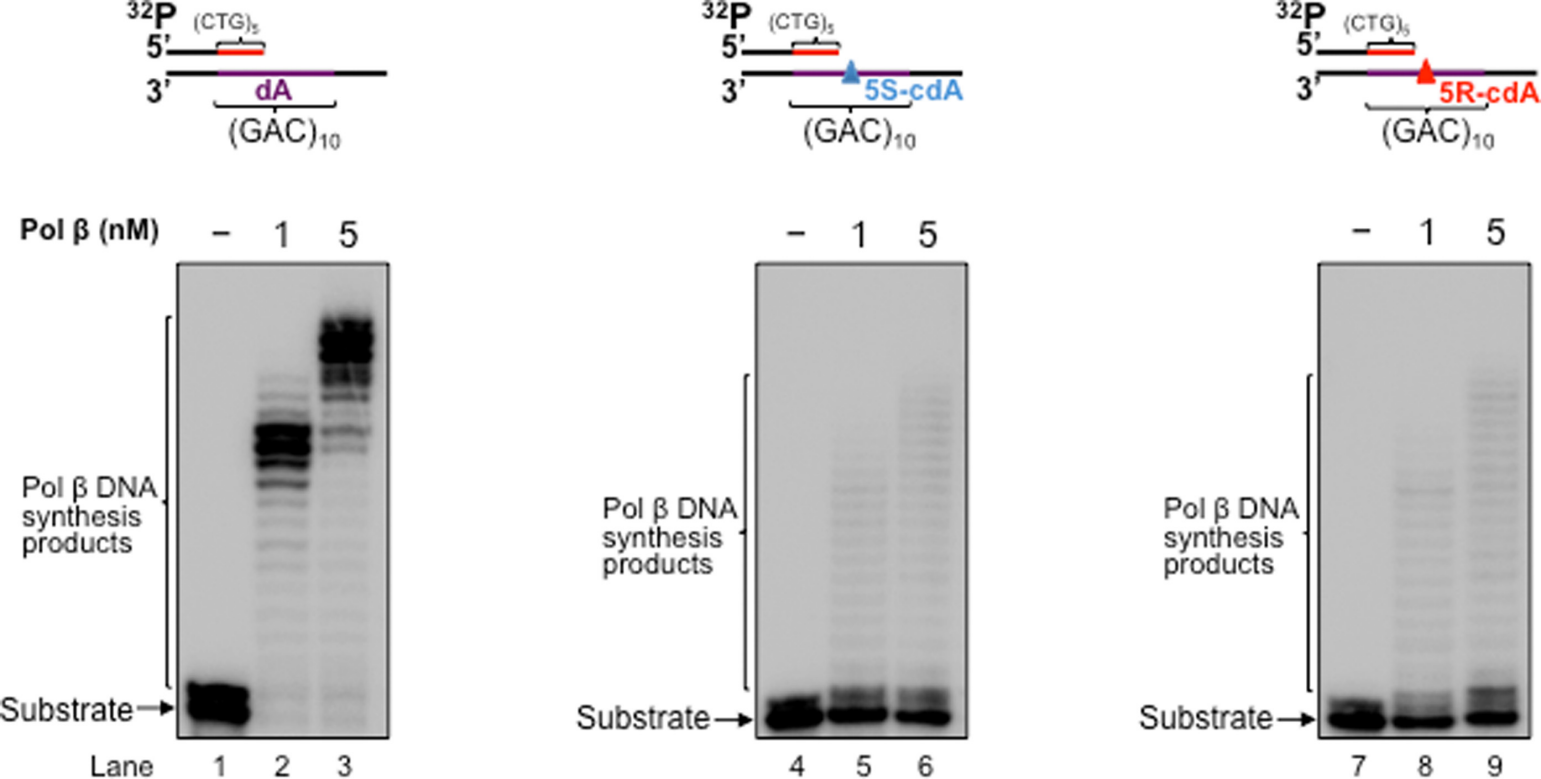
Pol β bypassed a cdA at an open template during DNA replication. Pol β DNA synthesis activity was examined with the substrates containing a dA, or 5S-cdA, or 5R-cdA with an upstream primer alone annealed with the template strand. Lanes 1, 4 and 7 correspond to substrates only. Lanes 2–3, 5–6 and 8–9 correspond to reaction mixtures with 1-nM and 5-nM pol β.

Pol β is an essential BER enzyme that performs single-nucleotide gap-filling synthesis during short-patch BER and multi-nucleotide gap-filling and strand displacement synthesis during long-patch BER (64). It mediates TNR expansion or deletion by inserting extra repeat units or by skipping over a portion of a template TNR hairpin during BER (27,43). Pol β can also perform lesion bypass synthesis during DNA leading and lagging strand synthesis (59,62) to facilitate Okazaki fragment maturation (59). During oxidative stress, multiple types of oxidized DNA base lesions, including 8-oxoGs and cdPus, are generated simultaneously in DNA *in vitro* and *in vivo* at a significant percentage (65,66). It is conceivable that the lesions can also occur in a TNR tract simultaneously in the DNA strands that are complementary to each other. Because cdPu lesions can readily accumulate in DNA, and an 8-oxoG on the complimentary strand can be efficiently removed for initiating the short-patch or long-patch BER, this allows pol β to encounter and bypass cdPu lesions located at various positions in a TNR tract during BER. Therefore, pol β could also bypass a cdA in a CAG repeat tract to facilitate BER and Okazaki fragment maturation, and this may alter the stability of CTG repeats. To test this possibility, we initially examined pol β lesion bypass synthesis activity with substrates containing a 1-nt gap opposite a dA (Figure 2A and B, lanes 2–3) or 5′S-cdA (Figure 2A and B, lanes 7–8) or 5′R-cdA (Figure 2A and B, lanes 12–13) embedded in the fifth CAG unit of a (CAG)_10_ repeat tract in the template strand. We found that pol β mainly inserted 1 nt to bypass a cdA lesion (Figure 2A and B, lanes 7–8 and 12–13), whereas it predominantly performed multi-nucleotide insertion to bypass a dA (Figure 2A and B, lanes 2–3) with substrates containing a downstream 5′-phosphorylated THF that mimics BER intermediates and substrates containing a downstream primer with a 5′-phosphate residue that mimics intermediates of Okazaki fragment maturation. This indicates that pol β bypassed a cdA lesion located in (CAG)_10_ repeats during BER and Okazaki fragment maturation. In the presence of FEN1, pol β DNA synthesis was increased with all of the substrates (Figure 2A and B, compare lanes 3, 8 and 13 with lanes 2, 7 and 12). The results indicate that pol β bypass of a cdA was enhanced by FEN1 cleavage of a downstream flap. Because previous studies showed that the efficiency of pol β DNA synthesis varied during BER of an oxidized base lesion that was located at different locations in a TNR tract (26,67), we further examined whether the position of a 1-nt gap relative to a template cdA in a (CAG)_10_ tract can modulate the efficiency of pol β lesion bypass synthesis by employing pol β DNA synthesis with the substrates containing a 1-nt gap located upstream or downstream of a template cdA (Figure 3). The results showed that with the substrates containing a 1-nt gap upstream of a cdA, pol β initially performed efficient DNA synthesis reaching to 1-nt prior to the lesion, and then inserted 7–15 nucleotides to bypass the lesion with a reduced efficiency (Figure 3A and B, lanes 7–8 and 12–13). However, with the substrates containing a 1-nt gap downstream of a cdA, pol β performed processive DNA synthesis (Figure 4A and B, lanes 7–8, lanes 12–13). This indicates that pol β bypassed a cdA with less efficiency than it synthesized DNA in the regions surrounding a cdA during BER and Okazaki fragment maturation. However, pol β DNA synthesis with these substrates was not affected by FEN1 (Figures 2–4, compare lanes 3, 8 and 13 with lanes 2, 7 and 12), indicating that FEN1 cleavage failed to facilitate pol β bypass of a cdA if a gap is upstream or opposite the lesion (Figures 2 and 3, lanes 3, 8 and 13). To further determine if pol β can bypass a cdA in an open template during DNA lagging strand synthesis, we examined pol β lesion bypass synthesis with a substrate containing a cdA located at the opened template strand without a downstream primer (Figure 5). We found that pol β bypassed the lesion with its multi-nucleotide DNA synthesis (Figure 5, lanes 5–9) and it performed processive DNA synthesis with an open template substrate containing a dA (Figure 5, lanes 2–3). The results indicate that pol β can also bypass a cdA in an open template.

### Pol β bypass of a cdA in a CAG repeat tract results in CTG repeat deletion during BER and Okazaki fragment maturation

A previous study showed that pol β bypass of a template AP site opposite a gap can result in DNA slippage and upstream primer realignment, resulting in sequence expansion or deletion (62). We reason that pol β bypass of a cdA may also induce TNR instability. To test this, we reconstituted BER or Okazaki fragment maturation with the substrates containing a dA or cdA in a (CAG)_10_ tract and determined the length change of the CTG repeats. We found that with the substrates containing a 1-nt gap opposite a dA or cdA, pol β directly bypassed the cdA during BER, leading to the formation of the full-length repaired product as well as a series of CTG repeat deletion products (Figure 2A, lanes 9 and 14). However, pol β DNA synthesis with the substrate containing a dA only resulted in the formation of the full-length repaired product that has the same length as the template (Figure 2A, lane 4). Similarly, we found that for the substrates containing a 1-nt gap opposite a cdA and a 5′-phoshorylated downstream primer, pol β bypass of the lesion resulted in the full-length product as well as a series of CTG repeat deletion products (Figure 2B, lanes 9 and 14). However, pol β DNA synthesis on the template with a dA only led to the production of the full-length product (Figure 2A and B, lane 4). To further determine if the position of a 1-nt gap relative to the lesion can also affect repeat length change, we reconstituted BER and Okazaki fragment maturation with substrates containing a 1-nt gap located upstream or downstream of a template dA or cdA (Figures 3 and 4, lanes 4, 9 and 14). The results showed that with a 1-nt gap located upstream of a template cdA, pol β lesion bypass led to both the full-length product and deletion products, mainly with one CTG deletion (Figure 3A and B, lanes 9 and 14). However, with a 1-nt gap located upstream of a template dA, pol β DNA synthesis only resulted in the formation of the full-length product (Figure 3A and B, lane 4). Pol β DNA synthesis with substrates containing a 1-nt gap located downstream of a template dA or cdA in a (CAG)_10_ repeat tract also only resulted in the full-length product (Figure 4A and B, lanes 4, 9 and 14). This indicates that CTG repeat deletions can be induced by pol β lesion bypass at the upstream or opposite a template cdA during BER and Okazaki fragment maturation. Our results indicate that the location of pol β lesion bypass synthesis relative to a template cdA in a (CAG)_10_ tract governs production of CTG repeat deletion products during BER and DNA replication, i.e. pol β lesion bypass synthesis at the upstream or opposite a template cdA results in CTG repeat deletion, whereas its DNA synthesis at the downstream of a cdA does not alter CTG repeat length. The results further suggest that a template cdA can induce looping out of CAG repeats at the template strand to create a loop structure during pol β lesion bypass, thereby promoting pol β to skip over the loop and causing CTG repeat deletion. To test this possibility, we then examined whether a cdA on the template strand can induce the formation of CAG repeat loop structures.

### A template cdA in a CAG repeat tract induces the formation of various sizes of loop structures that are skipped over by pol β

**Figure 6.**
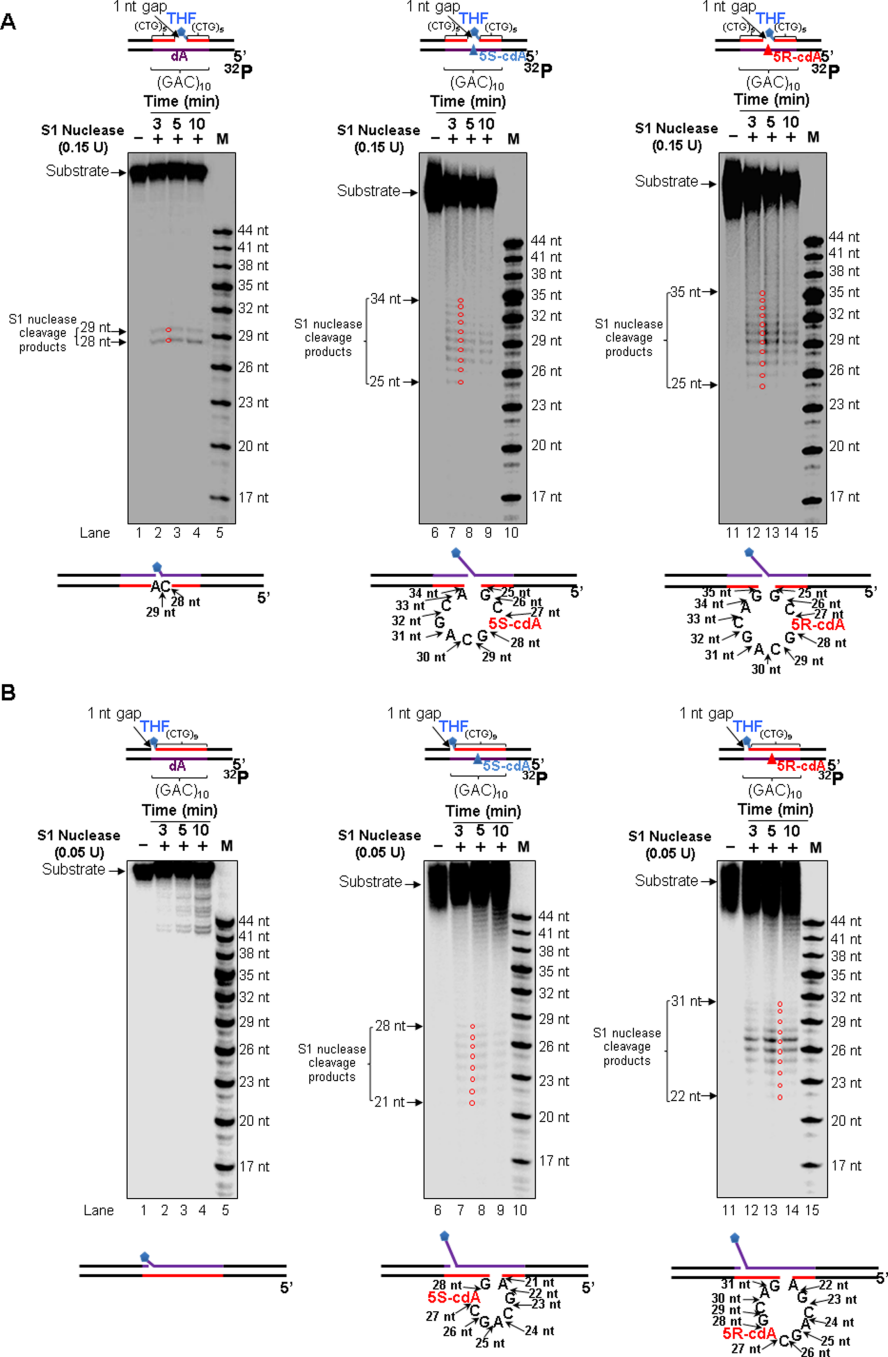
A template cdA induced the formation of a loop on a gapped DNA during pol β lesion bypass. Formation of a loop in the template strand of the substrates with a template dA, 5′S-cdA or 5′R-cdA in (CAG)_10_ repeats opposite (**A**) or downstream (**B**) of a 1-nt gap was probed in the absence of pol β. In all panels, substrates were radiolabeled at the 5′-end of its template strand. The substrates were incubated with 0.15 units (A) or 0.05 units (B) of S1 Nuclease at 3-, 5-, and 10-min time intervals (lanes 2–4, 7–9 and 12–14). Lanes 1, 6 and 11 represent the undigested substrate, whereas lanes 5, 10 and 15 represent size markers (M). For all the experiments, 25 nM of substrate was used. Arrows and circles indicate the major S1 Nuclease digestion products.

Previous studies have shown that during BER of oxidative DNA damage in a TNR tract, both the damaged and template strand can form hairpin structures (26,27). A hairpin formed on the damaged strand leads to TNR expansion, and a template hairpin results in TNR deletion (26,27). Interestingly, it has been found that formation of a cyclo-ring that links the C5 of the 2′-deoxyribose and the C8 of a purine results in a sugar pucker that is energetically unfavorable. This can then further induce DNA backbone distortion causing twisting of double-strand DNA (12). The distortion created by a cdPu in a TNR tract could promote the formation of secondary structures such as hairpins. Thus, the presence of a cdA in the template strand of a TNR tract could facilitate the formation of a hairpin or loop during pol β lesion bypass and promote pol β skip-over of the template hairpin or loop resulting in TNR deletion. To test this possibility, we initially examined the formation of a hairpin/loop in an intact double-strand DNA using S1 Nuclease, a single strand DNA specific nuclease (68) (Supplementary Figure S1). The results showed that S1 Nuclease resulted in no cleavage products with the double-strand DNA substrates with a template dA or a cdA (Supplementary Figure S1, lanes 2–3, 6–7 and 10–11). This indicates that cdA failed to induce the formation of any hairpin/loop structure in an intact double-strand DNA. To further determine whether a cdA may induce the formation of a hairpin/loop during BER, we examined S1 Nuclease cleavage on the template strand of the substrates with a 1-nt gap and 5′-THF residue that was opposite or upstream of a dA or a cdA (Figure 6). For the substrates with a 1-nt gap opposite a dA, S1 Nuclease cleavage resulted in the products with 28 nt and 29 nt (Figure 6A, lanes 2–4), indicating that the enzyme cleavage occurred opposite the 1-nt gap, and no hairpin/loop formed in the template strand. However, for the template strand with a 5′S-cdA or 5′R-cdA, the enzyme cleavage resulted in products with 25–34 nt and 25–35 nt indicating the formation of a (CAG)_3_ and (CAG)_4_ repeat loop containing a cdA (Figure 6A, lanes 7–9 and 12–14). To examine if pol β can skip over the CAG repeat loops during its bypass synthesis of a template cdA, we preincubated 1 nM pol β with the substrates for 30 min to allow the enzyme to perform its lesion bypass synthesis, and subsequently subjected the repair products to S1 Nuclease digestion. The results showed that S1 Nuclease on the template strand of the pol β lesion bypass products led to the same sizes of loops as the ones generated in the absence of pol β (Supplementary Figure S2A). This indicates that a (CAG)_3_ repeat loop containing a cdA still remained on the template strand after pol β lesion bypass synthesis, suggesting that pol β skipped over the loop structure. To examine if the location of a 1-nt gap may affect the formation of a CAG repeat loop induced by a cdA lesion during BER, we examined loop formation with the substrates containing a 1-nt gap located 14 nt upstream of a template dA or a cdA in the absence and presence of pol β (Figure 6B and Supplementary Figure S2B). We found that in the absence of pol β, S1 Nuclease cleavage on the substrate with a template dA only generated several cleavage products with a high molecular weight, indicating that S1 cleaves the 3′-side of the template strand which is not fully annealed with the upstream strand (Figure 6B, lanes 2–4). S1 Nuclease cleavage on the substrates with a template 5′S-cdA or 5′R-cdA led to products with 21–28 nt (Figure 6B, lanes 7–9) and products with 22–31 nt (Figure 6B, lanes 12–14), respectively, indicating the formation of a (CAG)_3_ and (CAG)_4_ repeat loop. S1 Nuclease cleavage on the lesion bypass products resulting from pol β lesion bypass led to products with 20–30 nt and products with 22–36 nt (Supplementary Figure S2B, lanes 7–9 and lanes 12–14), indicating the formation of a (CAG)_4_ and (CAG)_5_ loop, respectively. S1 Nuclease cleavage on the substrate with a template dA resulted in the same size of high molecular weight products as it did in the absence of pol β (Supplementary Figure S2B, lanes 2–4). These results indicate that a template cdA induced the formation of a CAG repeat loop that was skipped over by pol β during BER. To further determine if pol β can also skip over a CAG loop on the template strand during Okazaki fragment maturation, we probed loop formation in the substrates containing a 1-nt gap at different locations, along with a 5′-phosphorylated downstream primer (Supplementary Figure S3). The results showed that a (CAG)_3_ or (CAG)_4_ repeat loop in the template strand was also induced by a cdA (Supplementary Figure S3A and C), and the loop was also sustained after pol β lesion bypass synthesis (Supplementary Figure S3B and D). Thus, our results demonstrate that a cdA can induce the formation of a CAG repeat loop structure that is skipped over by pol β via DNA synthesis during both BER and DNA replication.

### A template cdA stimulates FEN1 cleavage on CTG repeats during BER and Okazaki fragment maturation

**Figure 7.**
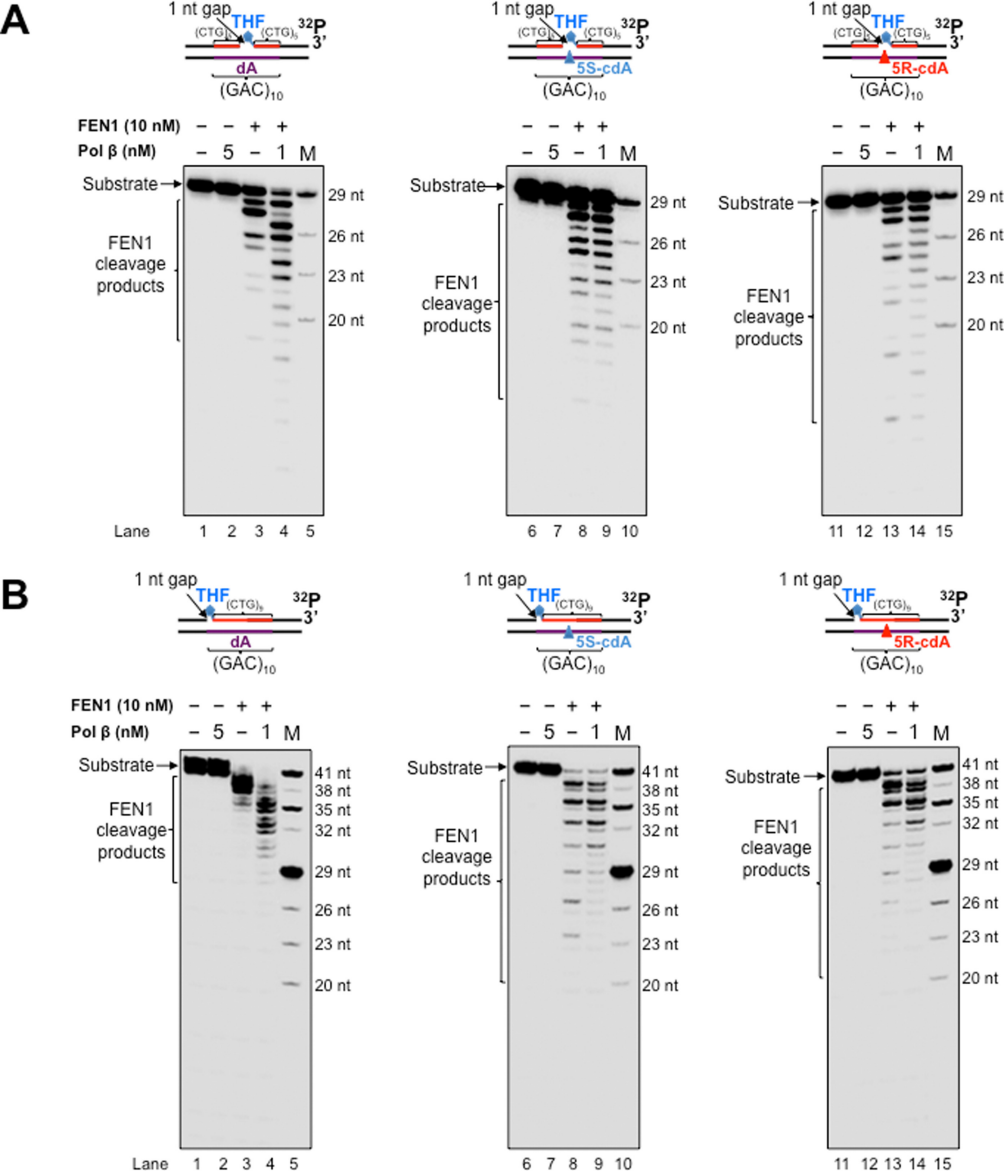
FEN1 flap cleavage during pol β bypass of a template cdA. FEN1 flap cleavage on the downstream strand of the substrates containing a 5′-THF residue with a 1-nt gap opposite (**A**) or upstream (**B**) of a template dA, 5′S-cdA or 5′R-cdA in a (CAG)_10_ repeat tract. Lanes 1, 6 and 11 represent substrates only. Lanes 2, 7 and 12 correspond to reaction mixtures with 5-nM pol β. Lanes 3–4, 8–9 and 13–14 correspond to reaction mixtures with 10-nM FEN1 in the absence of pol β and the presence of 1-nM pol β. Lanes 5, 10 and 15 correspond to synthesized size markers (M). Substrates were radiolabeled at the 3′-end of the 5′-THF containing strands.

FEN 1 is a core enzyme in both Okazaki fragment maturation and long-patch BER (69,70). FEN1 removes a 5′-flap generated by strand displacement synthesis mediated by replicative DNA polymerases such as pol δ/ϵ (70). FEN1 is also responsible for removing an oxidized or reduced deoxyribose phosphate that is subject to long-patch BER (64,71). In addition, FEN1 is a critical enzyme in preventing TNR expansion (42,46,72) by cleaving a TNR flap during Okazaki fragment maturation and BER in the loop region of a hairpin (50). However, FEN1 alternate flap cleavage activity can promote TNR expansion (43). Interestingly, coordination between pol β hairpin bypass synthesis and FEN1 flap cleavage can also result in TNR deletion during BER (27). It is conceivable that FEN1 coordinates with pol β to remove a 5′-flap generated by pol β bypass of a cdA, facilitating CTG repeat deletion. To test this, we initially determined if FEN1 cleavage on CTG repeats could be affected by a template cdA using the substrate with a 1-nt gap located opposite or upstream of a template cdA in the absence of pol β (Figure 7 and Supplementary Figure S4). We found that FEN1 flap cleavage with the substrates containing a template cdA generated longer products than its cleavage on the substrate with a template dA (compare lanes 8 and 13 with lane 3 of Figure 7A and B and Supplementary Figure S4A and B). This indicates that cdA facilitated FEN1 cleavage on longer flaps. This further suggests that cdA induced the formation of an intermediate containing a template loop with a longer flap that was efficiently cleaved by FEN1. However, in the presence of pol β DNA synthesis, a template cdA failed to exhibit the stimulatory effect on FEN1 flap cleavage with the substrates (compare lanes 9 and lanes 14 with lane 4 of Figure 7A and B and Supplementary Figure S4A and B). In contrast, pol β (1 nM) stimulated FEN1 cleavage on the substrates with a template dA (lane 4 of Figure 7A and B and Supplementary Figure S4A and B), indicating that pol β DNA synthesis created a CTG repeat flap for FEN1 cleavage. The results suggest that a template cdA induced the formation of a long CTG flap prior to FEN1 cleavage, presumably by distorting the DNA backbone of the template.

## DISCUSSION

**Figure 8.**
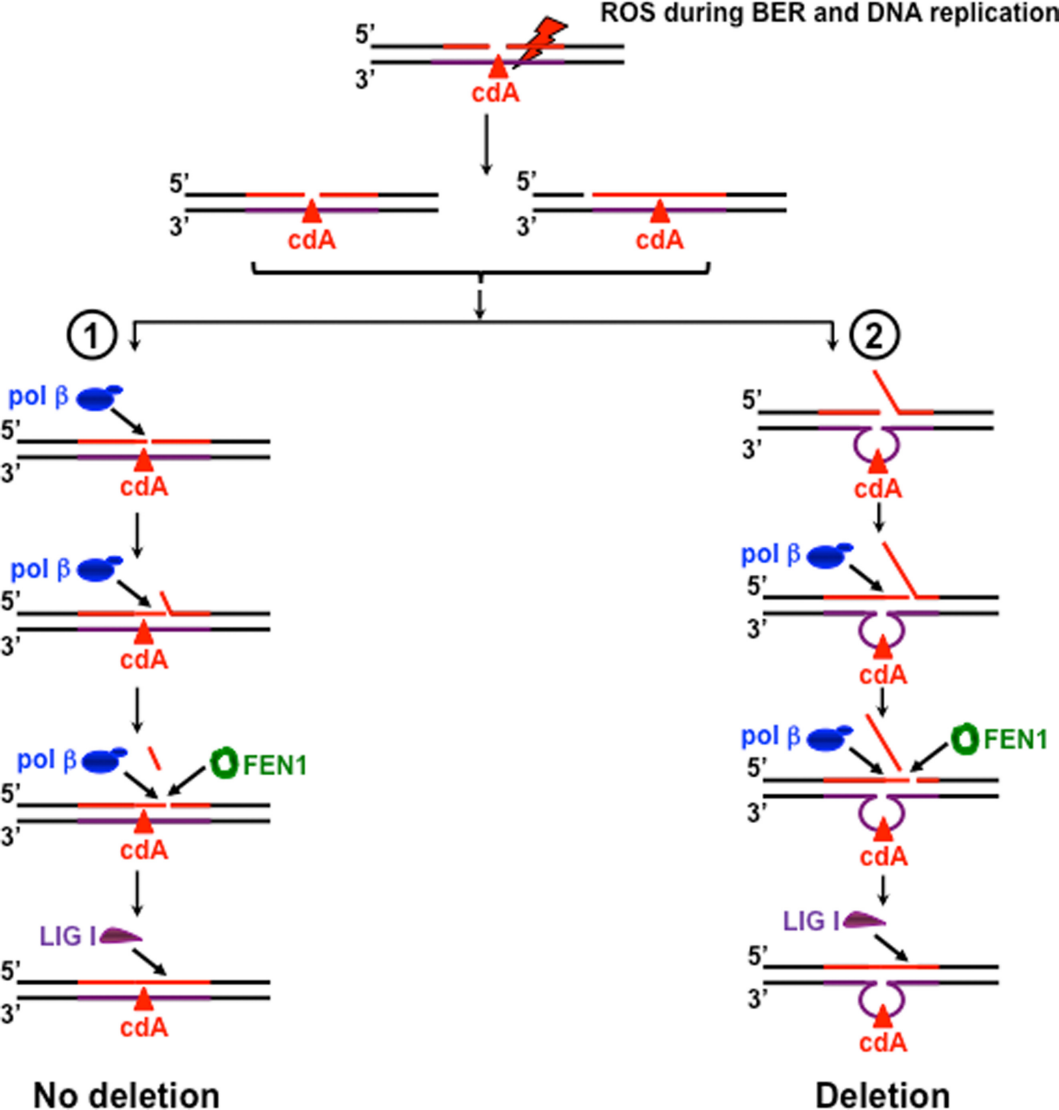
CTG repeat deletion via pol β bypass of a template cdA during BER and DNA replication. ROS generated by endogenous sources and radiation during BER and Okazaki maturation induces the production of a cdA on the template strand of a TNR tract located opposite or downstream of a gap. In one scenario, pol β can directly bypass the lesion leaving a nick for DNA LIG I to seal, resulting in no repeat deletion product (sub-pathway (1)). In another scenario, a template cdA can result in the formation of TNR loops of varying sizes that are located opposite or downstream of a 1-nt gap (sub-pathway (2)). This subsequently results in the formation of an intermediate with a template loop and a 5′-flap. Pol β then skips over the loop to perform lesion bypass synthesis. Subsequently, FEN1 cleaves the downstream flap leaving a nick for DNA LIG I to seal, resulting in small CTG repeat deletion products (sub-pathway (2)).

In this study, we provide the first evidence that pol β can bypass a template cdA located in a CAG repeat tract by skipping over a CAG repeat loop during BER and Okazaki fragment maturation (Figures 2 and 3). This subsequently results in CTG repeat deletions of varying sizes (Figures 2 and 3). Further characterization of DNA structures showed that a template cdA induced the formation of a CAG repeat loop that contained the lesion (Figure 6). Interestingly, we found that the loop still existed after pol β lesion bypass synthesis (Supplementary Figure S2), indicating that pol β skipped over the loop to bypass a cdA. Furthermore, we discovered that a template cdA also stimulated FEN1 cleavage on long flaps (lanes 8 and 13 of Figure 7A and B and Supplementary Figure S4A and B), suggesting the formation of an intermediate with a template loop and a long 5′-flap that can be efficiently cleaved by FEN1. Our results support a model in which a cdA in a TNR template that is located opposite or downstream of a 1-nt gap can be directly bypassed by pol β during BER and Okazaki fragment maturation. This would lead to a nicked DNA for DNA LIG I to seal, resulting in no repeat deletion (Figure 8, sub-pathway 1). On the other hand, a template cdA can induce the formation of a template loop that is located opposite or downstream of a 1-nt gap (Figure 8, sub-pathway 2). Subsequently, the loop promotes dissociation of the downstream strand from its template leading to the formation of an intermediate with a loop and a 5′-flap. FEN1 removes the 5′-flap, shortening the repeats and causing repeat deletion (Figure 8, sub-pathway 2).

Our results provide the first evidence that a cdA can induce TNR deletion by inducing the formation of various sizes of loop structures with the lesion in the template strand of a TNR repeat tract. This allows pol β to bypass the lesion by skipping over the loop structures. Moreover, our results suggest that formation of a loop on the template strand leads to dissociation of the downstream strand from its template strand creating a 5′-flap that is removed by FEN1 flap cleavage, thereby resulting in TNR deletion. Thus, our study demonstrates a novel pathway for cdPu lesions to induce TNR deletion via a unique pol β lesion bypass during DNA replication and BER. Our study further suggests that the unique lesion bypass of pol β could also mediate the instability of other repeated sequences such as dinucleotide repeats induced by a cdPu lesion. It is of interest to elucidate a pathway for cdPu-induced dinucleotide repeat instability during DNA metabolism.

In this study, we have discovered that CTG repeat deletion induced by a cdA via pol β lesion bypass can be modulated by the position of a gap relative to the location of the lesion. We found that CTG repeat deletion occurred only when a gap was located upstream or opposite a template cdA (Figures 2 and 3). A gap that was located downstream of the lesion failed to alter the stability of the repeats (Figure 4). This indicates that the formation of a template TNR loop downstream of a gap or single-strand break is essential for repeat deletion. Because repeat deletion is mediated by pol β skip-over of a template loop and FEN1 cleavage of repeats, this further suggests that a template TNR loop downstream of a single-strand break can promote pol β to skip over template CAG repeats and create a 5′-TNR flap that can be removed by FEN1. This subsequently shortens TNR tracts resulting in repeat deletion.

cdPu lesions create a blockage for DNA synthesis (13). During DNA replication, pol δ fails to bypass the lesion in the presence of proliferating cell nuclear antigen (PCNA) (13), indicating that replication polymerases cannot bypass a cdPu lesion by coordinating with replication cofactors. Because pol β can actively participate in lesion bypass during DNA replication by switching with pol δ or pol ϵ to bypass an abasic site (59,62), it could also play a crucial role in bypassing a cdPu lesion during DNA replication. Our results indicate that bypass of a cdA in the genome is also a challenge for pol β lesion bypass (Figures 2 and 3). This forces pol β to adopt a unique manner to bypass the lesion by skipping over a loop structure resulting in TNR deletion. It is conceivable that cofactors that can help pol β to directly bypass a cdA lesion may facilitate maintenance of TNR stability. It was reported that PCNA and replication protein A (RPA) can stimulate pol β bypass of an AP site during Okazaki fragment maturation (59). In addition, pol β bypass of an AP site can be facilitated by FEN1 (59). FEN1 and RPA can also coordinate with pol β to promote its strand displacement synthesis after pol β bypasses an AP site during Okazaki fragment maturation (59). Thus, it is of interest to determine if PCNA, FEN1 and RPA may help pol β to bypass a cdA directly sustaining TNR repeat stability.

Employing synthesized oligonucleotide substrates that mimic the intermediates generated during Okazaki fragment maturation and BER with a gap located at different locations relative to a cdA in the (CAG)_10_-containing template strand, we identified the formation of a small CAG repeat loop structure that was induced by a cdA. This indicates that cdA-induced loop structures underlie CTG repeat deletion. Thus, the substrates used in our study helped define a unique mechanism for a cdPu lesion to cause TNR deletion via pol β skip-over of a loop structure. Our observation of pol β skip-over of various sizes of CAG repeat loops induced by a template cdA further suggests that the stability of loop structures is the key to regulate the sizes of CTG repeat deletion products. Thus, BER and replication proteins that can modulate the stability of loop structures could modulate the size of TNR deletions. For example, mismatch repair proteins MSH2/MSH3 that can bind and stabilize a hairpin/loop (45) may facilitate pol β skip-over of a TNR loop on the template strand, promoting TNR deletion. In contrast, DNA replication and repair cofactors that can destabilize or disrupt loop structures, such as DNA helicases, may facilitate pol β to directly bypass a cdA, sustaining repeat stability. For example, Werner syndrome protein and Bloom syndrome protein can unwind a template TNR hairpin (73,74). It is possible that these proteins can facilitate pol β direct bypass of a cdA, by removing a repeat loop. The coordination between pol β and DNA helicases in preventing TNR instability remains to be elucidated.

FEN1 flap cleavage plays an essential role in modulating TNR expansion and deletion. Our results indicate that FEN1 cleavage on a 5′-flap induced by a cdA plays a crucial role in mediating repeat deletion. Thus, proteins that stimulate FEN1 activity may also promote TNR deletion. For example, PCNA can stimulate FEN1 flap cleavage by interacting with the enzyme (75). A 5′-3′ exonuclease, exonuclease I, and a helicase/endonuclease, Dna2, can facilitate FEN1 cleavage of TNRs by shortening a long flap (76,77). These enzymes could also play a role in promoting TNR deletion during pol β bypass of a cdA in a TNR tract, and their roles in modulating TNR instability induced by cdPu lesions need to be elucidated.

Previous studies showed that TNR expansion and deletion exhibits a length dependency. Here, we have demonstrated that a cdA base lesion located in a (CAG)_10_ repeat tract can lead to repeat deletion by inducing a small loop in the template strand. It is conceivable that a cdA lesion located in a long TNR tract can induce the formation of a large and more stable loop or hairpin in the template strand. This would facilitate pol β skip-over of the hairpin or loop, resulting in large TNR deletions (27). Moreover, because multiple cdA lesions may be generated in the same TNR tract, this may cause a large distortion of DNA structure that leads to formation of a large loop or hairpin or multiple loops and hairpins in the template, resulting in a large TNR deletion via pol β loop or hairpin bypass synthesis during DNA replication and BER.

In summary, in this study we have discovered a novel pathway for TNR deletion via pol β bypass of an oxidized DNA base lesion, cdA, during BER and DNA replication. We have demonstrated that a cdA can induce the formation of TNR loops of varying sizes on the template strand. Pol β can bypass the lesion by skipping over the loop structure, leading to repeat deletion. Simultaneously, formation of the loop on the template strand creates a 5′-flap that can be efficiently cleaved by FEN1. Our results reveal that pol β skip-over of a cdA coordinates with FEN1 flap cleavage to mediate TNR deletion.

## SUPPLEMENTARY DATA

Supplementary Data are available at NAR Online.

SUPPLEMENTARY DATA
