# Supplementary material for: A 5′, 8-cyclo-2′-deoxypurine lesion induces trinucleotide repeat deletion via a unique lesion bypass by DNA polymerase β
Source: Nucleic Acids Res. 2014 Nov 26;42(22):13749–63. doi: 10.1093/nar/gku1239 (PMC4267656; doi:10.1093/nar/gku1239)
Supplement: SUPPLEMENTARY DATA [file supp_42_22_13749__index.html]

A 5′, 8-cyclo-2′-deoxypurine lesion induces trinucleotide repeat deletion via a unique lesion bypass by DNA polymerase β — A 5′, 8-cyclo-2′-deoxypurine lesion induces trinucleotide repeat deletion via a unique lesion bypass by DNA polymerase β — SUPPLEMENTARY DATA 

# A 5′, 8-cyclo-2′-deoxypurine lesion induces trinucleotide repeat deletion via a unique lesion bypass by DNA polymerase β

## SUPPLEMENTARY DATA

**Files in this Data Supplement:**

- SUPPLEMENTARY DATA
