# Supplementary material for: A 5′, 8-cyclo-2′-deoxypurine lesion induces trinucleotide repeat deletion via a unique lesion bypass by DNA polymerase β
Source: Nucleic Acids Res. 2014 Nov 26;42(22):13749–63. doi: 10.1093/nar/gku1239 (PMC4267656; doi:10.1093/nar/gku1239)
Supplement: SUPPLEMENTARY DATA [file supp_gku1239_nar-02572-f-2014-File010.pdf]

Table S1 Oligonucleotide sequence

Oligonucleotides	nt	Sequence (5’ –3’)
Downstream		
D1	27	p**F** G CTG CTG CTG CTG TA CGG ATG CTA GAT
D2	41	p**F** CTG CTG CTG CTG CTG CTG CTG CTG CTG TA CGG ATG CTA GAT
D3	14	p**F** TA CGG ATG CTA GAT
D4	27	pG CTG CTG CTG CTG TA CGG ATG CTA GAT
D5	41	pCTG CTG CTG CTG CTG CTG CTG CTG CTG TA CGG ATG CTA GAT
D6	14	pTA CGG ATG CTA GAT
D7	22	pTG CTG CTG TA CGG ATG CTA GAT
D8	26	pCTG CTG CTG CTG TA CGG ATG CTA GAT
Template		
T1	58	ATC TAG CAT CCG TAC AGC AGC AGC AGC AGC AGC AGC AGC AGC AGT ACG TAG ACT TAC T
T2	58	ATC TAG CAT CCG TAC AGC AGC AGC AGC 5’S-cdAGC AGC AGC AGC AGC AGT ACG TAG ACT TAC T
T3	58	ATC TAG CAT CCG TAC AGC AGC AGC AGC 5’R-cdAGC AGC AGC AGC AGC AGT ACG TAG ACT TAC T
Upstream		
U1	30	AGT AAG TCT ACG TA CTG CTG CTG CTG CTG C
U2	16	AGT AAG TCT ACG TA CT
U3	43	AGT AAG TCT ACG TA CTG CTG CTG CTG CTG CTG CTG CTG CTG CT
U4	26	AGT AAG TCT ACG TA CTG CTG CTG CTG

F: tetrahydrofuran, THF

Fig. S1

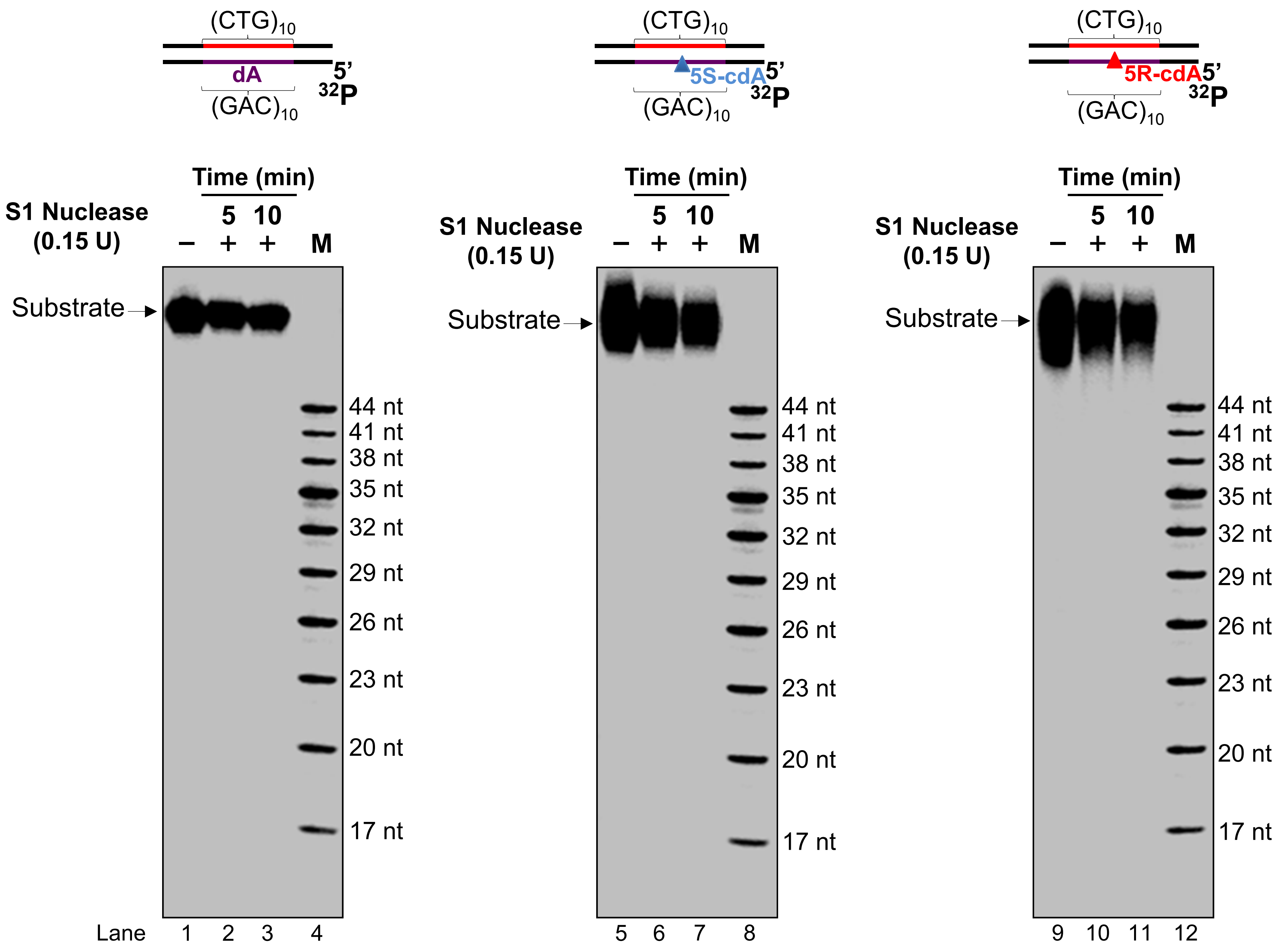
(CTG)10
dA
5'
32P
(GAC)10
5S-cdA
5R-cdA
Time (min)
S1 Nuclease
(0.15 U)
5 10
– + + M
Substrate
44 nt
41 nt
38 nt
35 nt
32 nt
29 nt
26 nt
23 nt
20 nt
17 nt
Lane 1 2 3 4
5 6 7 8
9 10 11 12

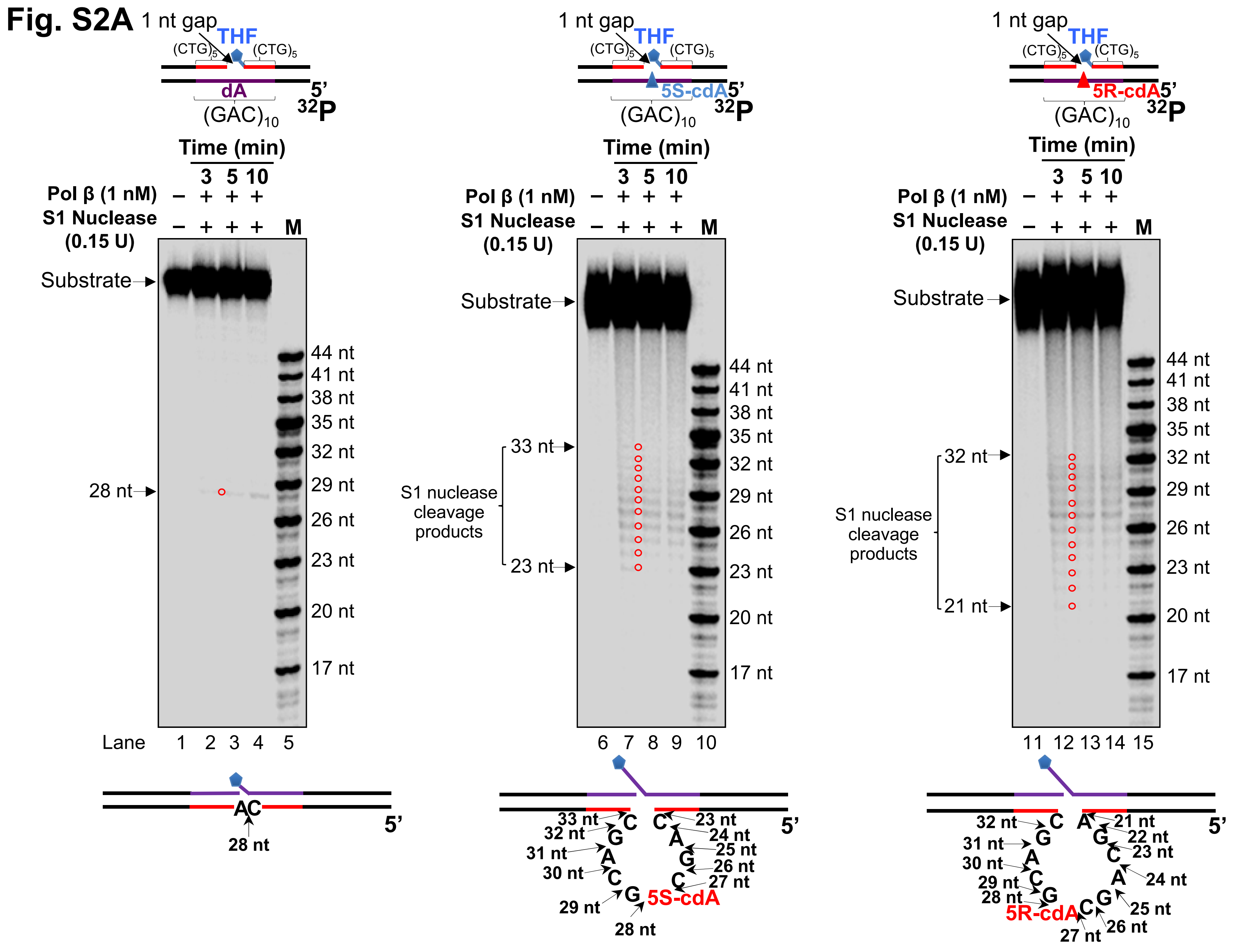

Fig. S2A
1 nt gap
THF
(CTG)5
dA
5'
32P
(GAC)10
5S-cdA
5R-cdA
Time (min)
3 5 10
Pol β (1 nM)
S1 Nuclease (0.15 U)
M
Substrate
28 nt
44 nt
41 nt
38 nt
35 nt
32 nt
29 nt
26 nt
23 nt
20 nt
17 nt
33 nt
21 nt
S1 nuclease cleavage products
Lane 1 2 3 4 5 6 7 8 9 10 11 12 13 14 15

Fig. S2B

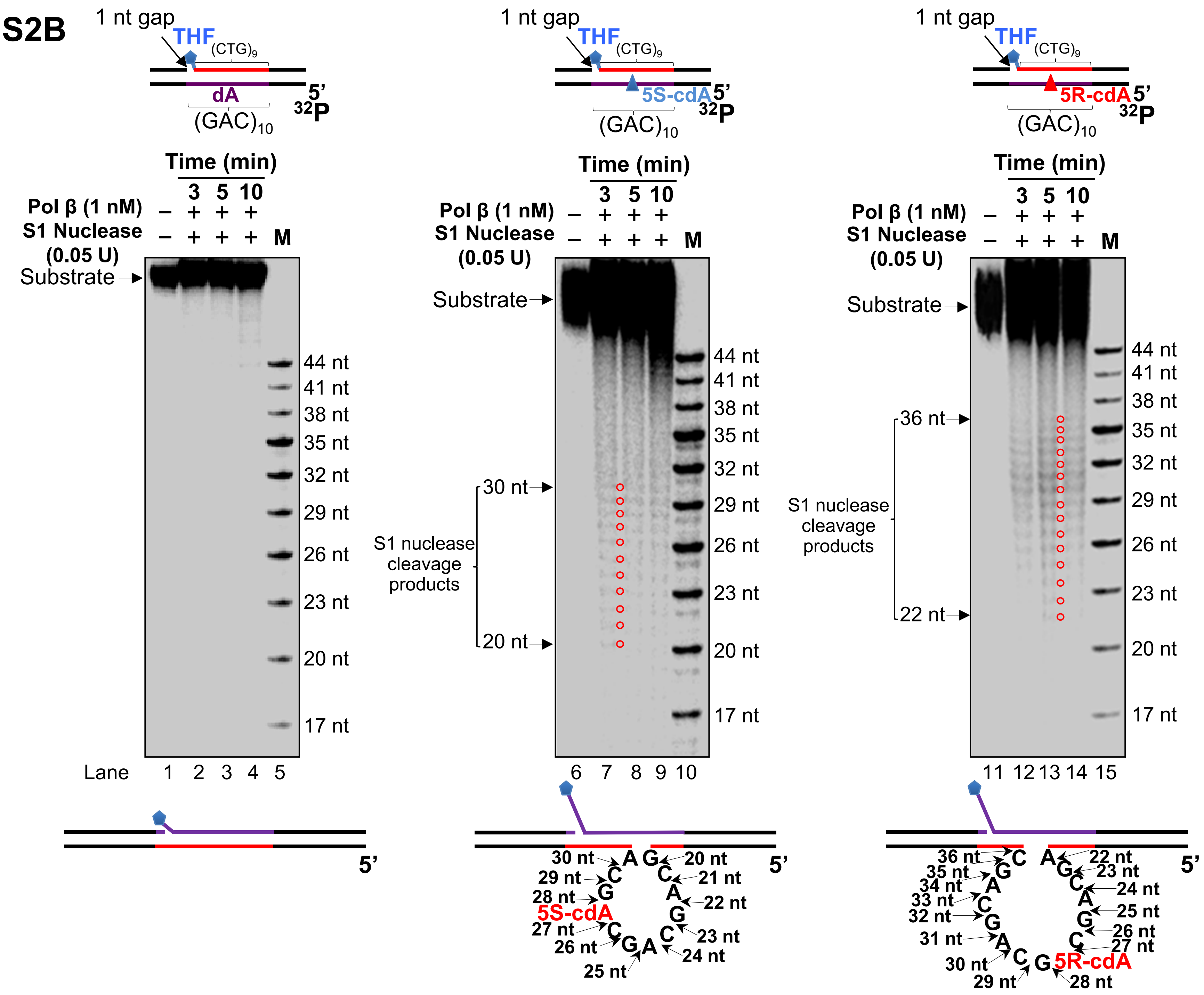

1 nt gap
THF
(CTG)9
dA
(GAC)10
5'
32P
5S-cdA
5R-cdA
Time (min)
3 5 10
Pol β (1 nM) – + + +
S1 Nuclease (0.05 U) – + + + M
Substrate
44 nt
41 nt
38 nt
35 nt
32 nt
29 nt
26 nt
23 nt
20 nt
17 nt
30 nt
20 nt
36 nt
22 nt
S1 nuclease cleavage products
Lane 1 2 3 4 5
6 7 8 9 10
11 12 13 14 15

Fig. S3A

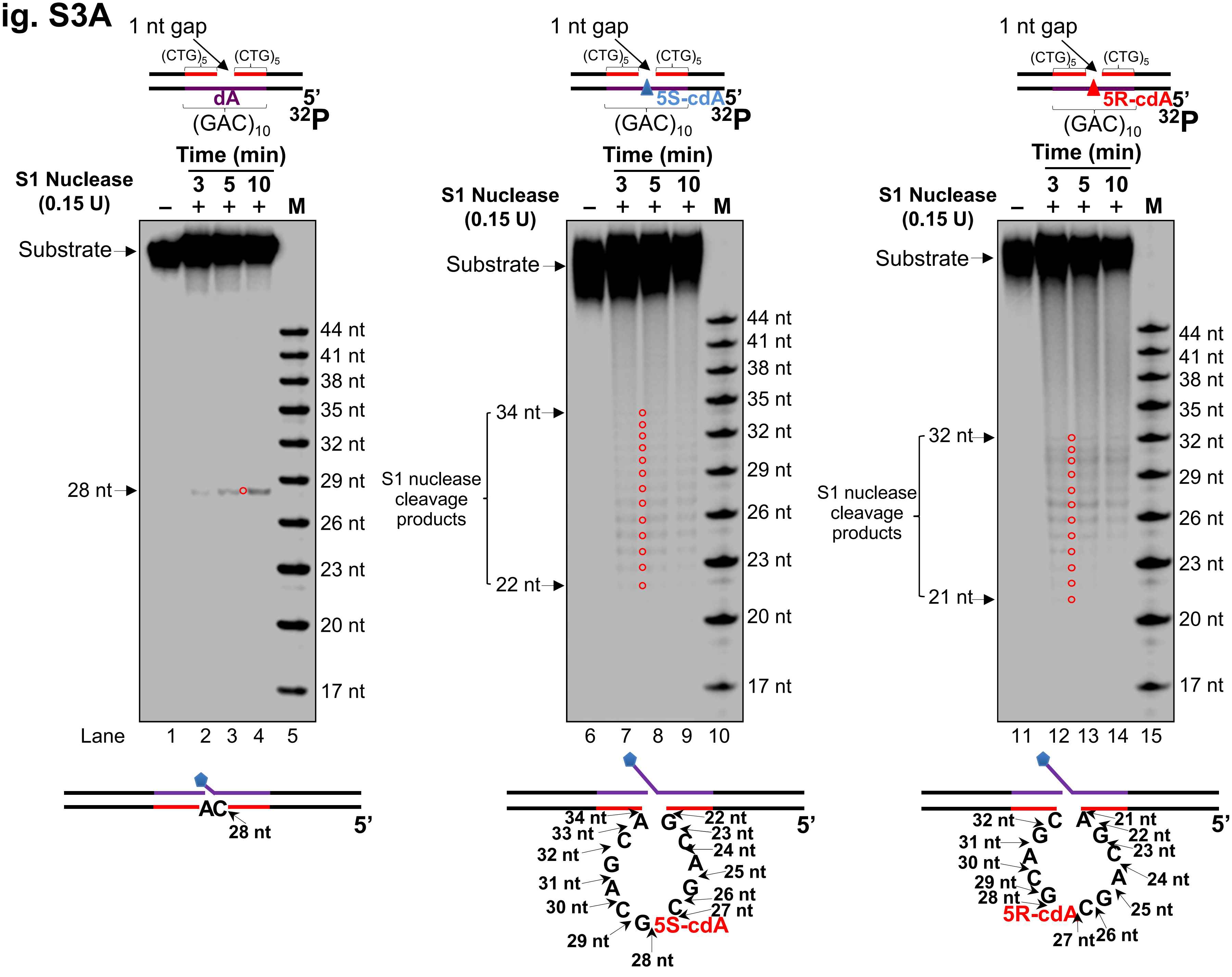
1 nt gap
(CTG)5
(CTG)5
dA
5'
32P
(GAC)10
5S-cdA
5R-cdA
Time (min)
S1 Nuclease
(0.15 U)
3 5 10
– + + + M
Substrate
28 nt
34 nt
22 nt
32 nt
21 nt
S1 nuclease cleavage products
44 nt
41 nt
38 nt
35 nt
32 nt
29 nt
26 nt
23 nt
20 nt
17 nt
Lane 1 2 3 4 5
6 7 8 9 10
11 12 13 14 15

Fig. S3B

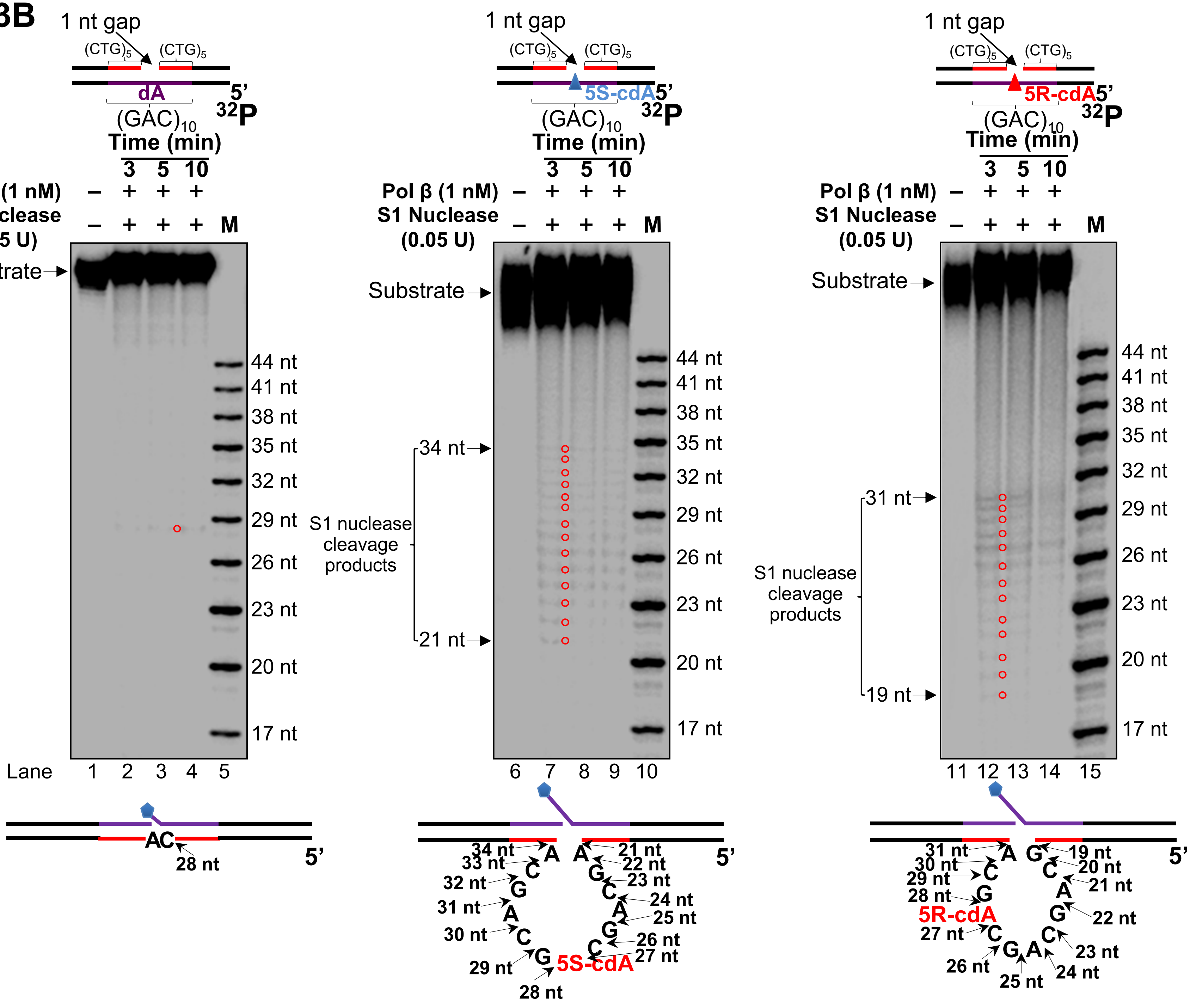

1 nt gap
(CTG)5
(CTG)5
dA
5'
32P
(GAC)10
Time (min)
3 5 10
Pol β (1 nM)
S1 Nuclease (0.05 U)
M
Substrate
44 nt
41 nt
38 nt
35 nt
32 nt
29 nt
26 nt
23 nt
20 nt
17 nt
Lane
1 2 3 4 5
AC
28 nt
5S-cdA
34 nt
21 nt
S1 nuclease cleavage products
6 7 8 9 10
33 nt
32 nt
31 nt
30 nt
29 nt
28 nt
22 nt
23 nt
24 nt
25 nt
26 nt
27 nt
5R-cdA
31 nt
19 nt
11 12 13 14 15
30 nt
29 nt
28 nt
27 nt
26 nt
25 nt
24 nt
23 nt
22 nt
21 nt
20 nt
19 nt

Fig. S3C

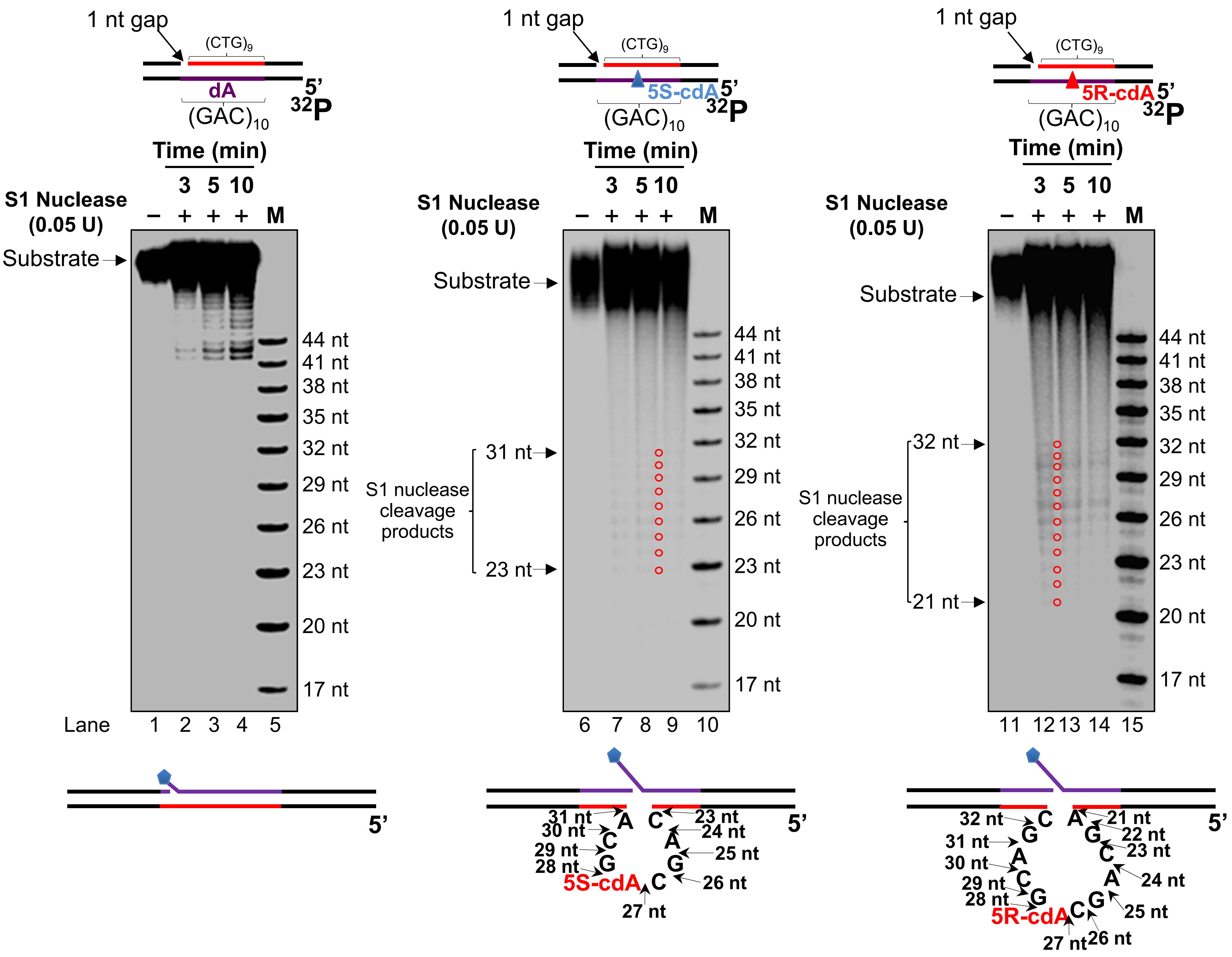

1 nt gap
(CTG)9
dA
(GAC)10
5'
32P
5S-cdA
5R-cdA
Time (min)
3 5 10
S1 Nuclease (0.05 U)
– + + + M
Substrate
44 nt
41 nt
38 nt
35 nt
32 nt
29 nt
26 nt
23 nt
20 nt
17 nt
31 nt
23 nt
21 nt
S1 nuclease cleavage products
Lane 1 2 3 4 5 6 7 8 9 10 11 12 13 14 15

Fig. S3D

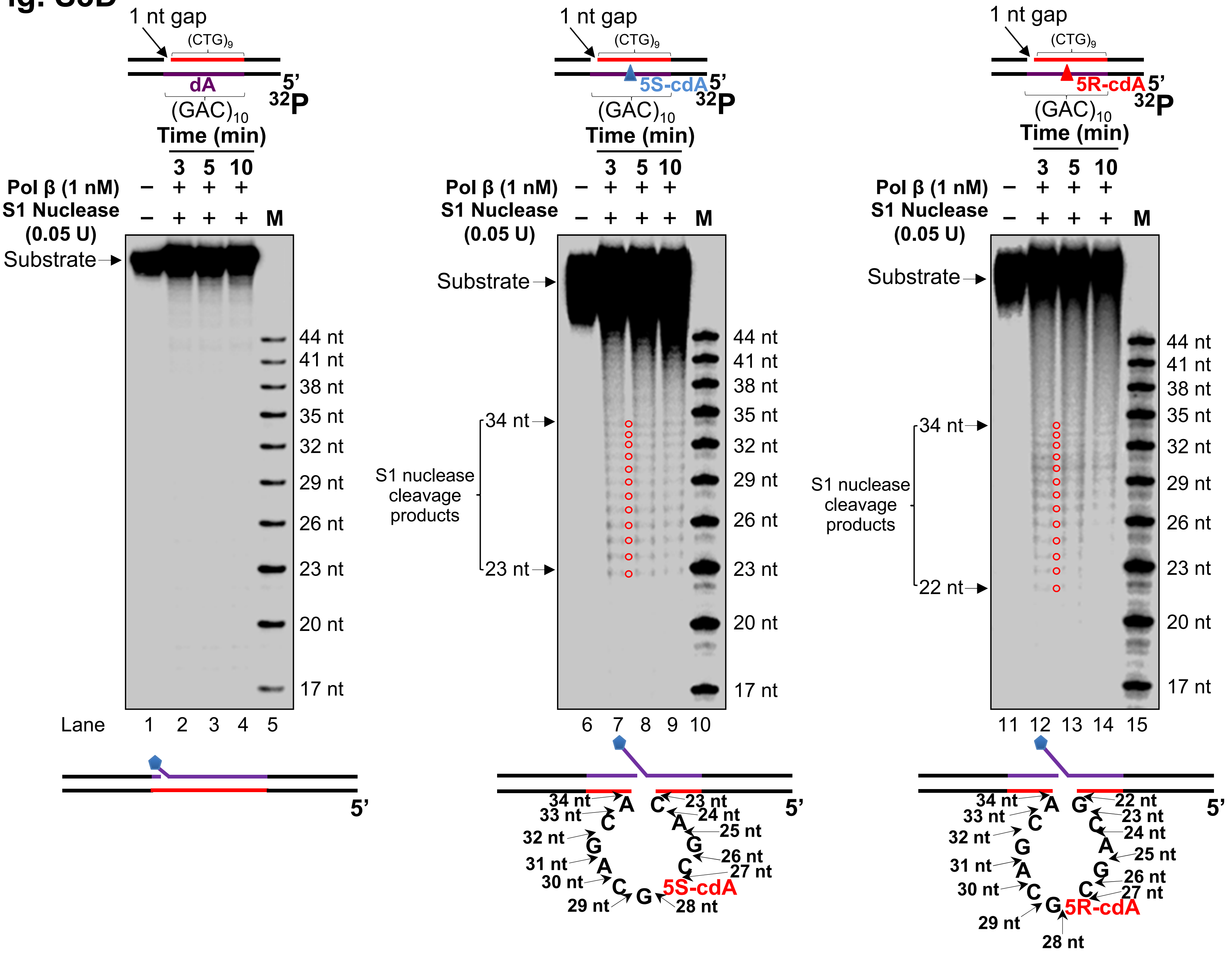
1 nt gap
(CTG)9
dA
5'
32P
(GAC)10
Time (min)
3 5 10
Pol β (1 nM) − + + +
S1 Nuclease (0.05 U) − + + + M
Substrate
44 nt
41 nt
38 nt
35 nt
32 nt
29 nt
26 nt
23 nt
20 nt
17 nt
Lane 1 2 3 4 5
5'
1 nt gap
(CTG)9
5S-cdA
5'
32P
(GAC)10
Time (min)
3 5 10
Pol β (1 nM) − + + +
S1 Nuclease (0.05 U) − + + + M
Substrate
34 nt
S1 nuclease cleavage products
23 nt
44 nt
41 nt
38 nt
35 nt
32 nt
29 nt
26 nt
23 nt
20 nt
17 nt
6 7 8 9 10
34 nt
33 nt
32 nt
31 nt
30 nt
29 nt
28 nt
27 nt
26 nt
25 nt
24 nt
23 nt
5S-cdA
5'
1 nt gap
(CTG)9
5R-cdA
5'
32P
(GAC)10
Time (min)
3 5 10
Pol β (1 nM) − + + +
S1 Nuclease (0.05 U) − + + + M
Substrate
34 nt
S1 nuclease cleavage products
22 nt
44 nt
41 nt
38 nt
35 nt
32 nt
29 nt
26 nt
23 nt
20 nt
17 nt
11 12 13 14 15
34 nt
33 nt
32 nt
31 nt
30 nt
29 nt
28 nt
27 nt
26 nt
25 nt
24 nt
23 nt
22 nt
5R-cdA
5'

Fig. S4A

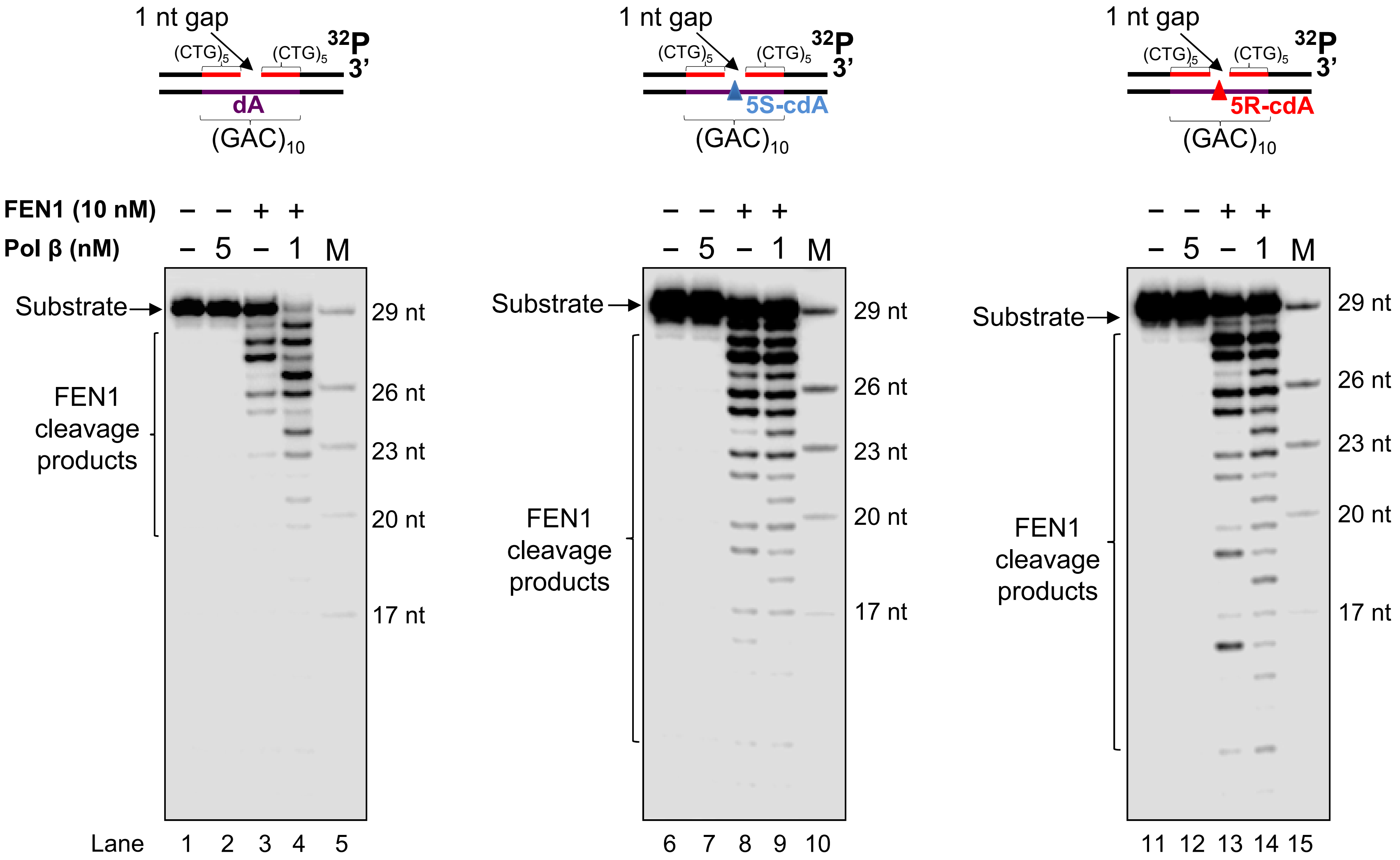

1 nt gap
(CTG)5
(CTG)5
32P
3'
dA
5S-cdA
5R-cdA
(GAC)10
FEN1 (10 nM) – – + + – – + + – – + +
Pol β (nM) – 5 – 1 M – 5 – 1 M – 5 – 1 M
Substrate
FEN1 cleavage products
29 nt
26 nt
23 nt
20 nt
17 nt
Lane 1 2 3 4 5 6 7 8 9 10 11 12 13 14 15

Fig. S4B

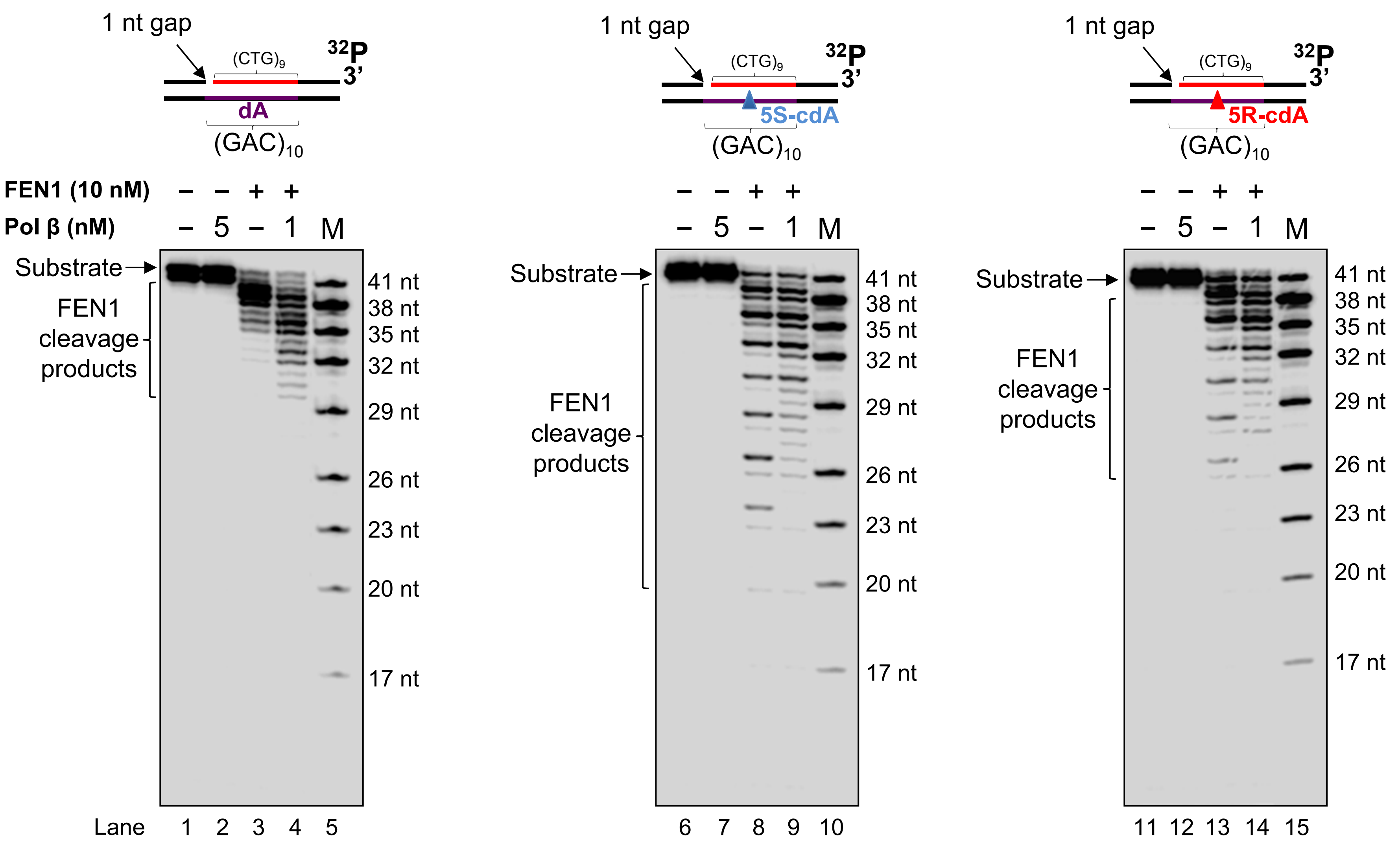
1 nt gap
(CTG)9
32P
3'
dA
(GAC)10
5S-cdA
5R-cdA
FEN1 (10 nM)
Pol β (nM)
– – + +
– 5 – 1 M
Substrate
FEN1 cleavage products
41 nt
38 nt
35 nt
32 nt
29 nt
26 nt
23 nt
20 nt
17 nt
Lane
1 2 3 4 5
6 7 8 9 10
11 12 13 14 15

Supplemental Data

Supplemental Figure Legends

Supplementary Figure S1. A template 5',8-cdA failed to induce the formation of a loop on an intact double-strand DNA. Formation of a loop in the template strand of the intact double-strand DNA substrates with a dA (left panel), 5'S-cdA (middle panel) or 5'R-cdA (right panel) in $(CAG)_{10}$ repeats was probed by S1 Nuclease digestion. Substrates were radiolabeled at the 5'-end of its template strand and incubated with 0.15 units of S1 Nuclease at 5- and 10-minute time intervals (lanes 2-3, 6-7 and 10-11). Lanes 1, 5 and 9 represent the undigested substrate. Lanes 4, 8 and 12 represent synthesized size markers (M). For all experiments, 25 nM of substrate was used. Arrows and circles indicate the major S1 Nuclease digestion products. The substrates are illustrated schematically above the gel.

Supplemental Figure S2. A 5',8-cdA induced loop structure was sustained after pol β lesion bypass synthesis during BER. The template strand of the substrates with a template dA (left panel), 5'S-cdA (middle panel) or 5'R-cdA (right panel) in $(CAG)_{10}$ repeats opposite**(A)** or downstream **(B)** of a 1-nt gap was radiolabeled at the 5'-end. The substrates were pre-incubated with 1 nM pol β for 30 min at 37 ℃ and subsequently subjected to S1 Nuclease digestion. The substrates were digested with 0.15 units **(A)** or 0.05 units **(B)** of S1 Nuclease at 3-, 5-and 10-minute time intervals (lanes 2-4, 7-9 and 12-14). Lanes 1, 6 and 11 represent the undigested substrate. Lanes 5, 10 and 15 represent synthesized size markers (M). For all experiments, 25 nM of substrate was used. Arrows and circles indicate the major S1 Nuclease digestion products. The substrates are illustrated schematically above the gel. A CAG loop deduced by the specific nuclease cleavage pattern, and the nuclease digestion sites are illustrated schematically below the gel.

Supplemental Figure S3. A 5',8-cdA induced loop structure was sustained after pol β lesion bypass synthesis during DNA replication. The template strand of the substrates with a 1-nt gap opposite to a template dA (left panel), 5'S-cdA (middle panel) or 5'R-cdA (right panel) in $(CAG)_{10}$ repeatswas radiolabeled at the 5'-end. The substrates were incubated with S1 Nuclease without pol β **(A)** or pre-incubated with 1 nM pol β for 30 min at 37 ℃ **(B)**. Substrates were incubated with 0.15 units of S1 Nuclease at 3-, 5- and 10-minute time intervals (lanes 2-4, 7-9 and 12-14). Formation of the template loop of the substrates with a 1-nt gap located upstream of a template dA (left panel), 5'S-cdA (middle panel) or 5'R-cdA (right panel) in $(CAG)_{10}$ repeats was probed by 0.05 unit of S1 Nuclease. The substrates were incubated with S1 Nuclease without pol β **(C)** or pre-incubated with 1 nM pol β for 30 min at 37 ℃ **(D)**. The substrates were digested with 0.05 units of S1 Nuclease at 3-, 5-and 10-minute time intervals (lanes 2-4, 7-9 and 12-14). Lanes 1, 6 and 11 represent the undigested substrate. Lanes 5, 10 and 15 represent synthesized size markers (M). For all experiments, 25 nM of substrate was used. Arrows and circles indicate the major S1 Nuclease digestion products. The substrates are illustrated schematically above the gel. A CAG loop deduced by the specific nuclease cleavage pattern, and the nuclease digestion sites are illustrated schematically below the gel.

Supplemental Figure S4. FEN1 flap cleavage during pol β bypass of a template cdA. FEN1 flap cleavage on the downstream strand of the substrates containing a 5'-phosphate with a 1-nt gap opposite **(A)** or upstream **(B)** of a template dA (left panel), 5'S-cdA (middle panel) or 5'R-cdA (right panel) in a $(CAG)_{10}$ repeat tract. Lanes 1, 6 and 11 represent substrates only. Lanes 2, 7 and 12 correspond to reaction mixtures with 5 nM pol β. Lanes 3-4, 8-9 and 13-14 correspond to reaction mixtures with 10 nM FEN1 in the absence and presence of 1 nM pol β. Lanes 5, 10 and 15 correspond to synthesized size markers (M). Substrates were radiolabeled at the 3'-end of the 5'-THF containing strands.
